# Revisiting the Neural Basis of Acquired Amusia: Lesion Patterns and Structural Changes Underlying Amusia Recovery

**DOI:** 10.3389/fnins.2017.00426

**Published:** 2017-07-25

**Authors:** Aleksi J. Sihvonen, Pablo Ripollés, Antoni Rodríguez-Fornells, Seppo Soinila, Teppo Särkämö

**Affiliations:** ^1^Faculty of Medicine, University of Turku Turku, Finland; ^2^Cognitive Brain Research Unit, Department of Psychology and Logopedics, Faculty of Medicine, University of Helsinki Helsinki, Finland; ^3^Cognition and Brain Plasticity Group, Bellvitge Biomedical Research Institute (IDIBELL), L'Hospitalet de Llobregat Barcelona, Spain; ^4^Department of Cognition, Development and Education Psychology, University of Barcelona Barcelona, Spain; ^5^Poeppel Lab, Department of Psychology, New York University New York, NY, United States; ^6^Catalan Institution for Research and Advanced Studies, Institució Catalana de Recerca i Estudis Avançats (ICREA) Barcelona, Spain; ^7^Division of Clinical Neurosciences, Turku University Hospital and Department of Neurology, University of Turku Turku, Finland

**Keywords:** music, amusia, stroke, recovery, voxel-based morphometry, voxel-based lesion-symptom mapping

## Abstract

Although, acquired amusia is a common deficit following stroke, relatively little is still known about its precise neural basis, let alone to its recovery. Recently, we performed a voxel-based lesion-symptom mapping (VLSM) and morphometry (VBM) study which revealed a right lateralized lesion pattern, and longitudinal gray matter volume (GMV) and white matter volume (WMV) changes that were specifically associated with acquired amusia after stroke. In the present study, using a larger sample of stroke patients (*N* = 90), we aimed to replicate and extend the previous structural findings as well as to determine the lesion patterns and volumetric changes associated with amusia recovery. Structural MRIs were acquired at acute and 6-month post-stroke stages. Music perception was behaviorally assessed at acute and 3-month post-stroke stages using the Scale and Rhythm subtests of the Montreal Battery of Evaluation of Amusia (MBEA). Using these scores, the patients were classified as non-amusic, recovered amusic, and non-recovered amusic. The results of the acute stage VLSM analyses and the longitudinal VBM analyses converged to show that more severe and persistent (non-recovered) amusia was associated with an extensive pattern of lesions and GMV/WMV decrease in right temporal, frontal, parietal, striatal, and limbic areas. In contrast, less severe and transient (recovered) amusia was linked to lesions specifically in left inferior frontal gyrus as well as to a GMV decrease in right parietal areas. Separate continuous analyses of MBEA Scale and Rhythm scores showed extensively overlapping lesion pattern in right temporal, frontal, and subcortical structures as well as in the right insula. Interestingly, the recovered pitch amusia was related to smaller GMV decreases in the temporoparietal junction whereas the recovered rhythm amusia was associated to smaller GMV decreases in the inferior temporal pole. Overall, the results provide a more comprehensive picture of the lesions and longitudinal structural changes associated with different recovery trajectories of acquired amusia.

## Introduction

The perception and experience of music in the healthy brain is based on the functioning of a large-scale bilateral neural network comprising temporal, frontal, parietal, cerebellar, and subcortical areas (Schmithorst, 2005; Brattico et al., 2011; Alluri et al., 2012; Zatorre and Salimpoor, 2013; Koelsch, 2014). In amusia, the ability to perceive music is impaired by either abnormal brain development (congenital amusia) or brain tissue damage (acquired amusia). While congenital amusia is generally described as a deficit in processing pitch—arguably due to an impairment of pitch perception and/or pitch-specific short-term or working memory—the processing of musical rhythm, timbre, and emotions can be affected as well (Stewart et al., 2006; Marin et al., 2012; Tillmann et al., 2015, 2016; Peretz, 2016; Whiteford and Oxenham, 2017).

The majority of the neuroimaging studies examining defective music processing in the brain have been carried out on congenital amusia, a condition affecting 2–4% of the population (Kalmus and Fry, 1980; Henry and McAuley, 2010). In contrast, acquired amusia is a relatively common disorder after a middle cerebral artery (MCA) stroke, with incidence ranging from 35 to 69% (Ayotte et al., 2000; Schuppert et al., 2000; Särkämö et al., 2009; Sihvonen et al., 2016). Evidence derived from MRI morphometry studies, utilizing e.g., voxel-based morphometry (VBM; Ashburner and Friston, 2000), an automated method for analyzing gray matter and white matter differences between groups or across time, has implicated reduced white matter concentration in the right inferior frontal gyrus (IFG; Hyde et al., 2006; Albouy et al., 2013) and right superior temporal gyrus (STG; Albouy et al., 2013) in congenital amusics. Additionally, the cortex in both of these areas have been shown to be thicker in congenital amusic subjects than in controls (Hyde et al., 2007). However, the reported results have been contradictory regarding laterality of the observed effect: a recent study showed that congenital amusics had decreased gray matter volume (GMV) in the left IFG and STG with no differences observed in the right homologous areas (Mandell et al., 2007). Taken together, these findings suggest that congenital amusia may be a somewhat heterogeneous condition (Omigie et al., 2012).

The two types of amusia, acquired and congenital, may have partly distinct neural basis. Congenital amusia is a developmental deficit and thus impedes acquiring musical syntax (Stewart, 2008), whereas acquired amusia represents a shift from a normal to deficiently functioning music processing system caused by a brain lesion. Studying brain lesions and associated cognitive deficits is essential in uncovering crucial brain regions that are causally connected (Rorden and Karnath, 2004). This can be achieved by voxel-based lesion-symptom mapping (VLSM), which is an advanced MRI analysis method investigating the relationship between focal brain damage and behavioral data on a voxel-by-voxel basis (Bates et al., 2003). Compared to the traditional lesion-led or symptom-led approaches, VLSM allows both binary and continuous analyses and does not require patient grouping by lesion site. VLSM utilizes three-dimensional lesion maps formed from MRI images, and evaluates the presence or absence of lesion in each voxel to finally associate this information with the behavioral data.

Previous studies investigating the neural basis of acquired amusia have been limited to symptom-led and lesion-led studies of individual cases or small patient groups (Kester et al., 1991; Liegeois-Chauvel et al., 1998; Ayotte et al., 2000; Schuppert et al., 2000; Rosslau et al., 2015; for a review see Stewart et al., 2006). Recently, we utilized VLSM in a sample of 77 stroke patients from two Finnish cohorts (from Helsinki and Turku) to map the lesioned brain regions specifically associated with acquired amusia (Sihvonen et al., 2016). The results revealed that damage to the right STG, middle temporal gyrus (MTG), insula, and putamen form the crucial neural substrate for acquired amusia. In addition, we performed longitudinal VBM analyses on the Helsinki cohort, which showed that patients with non-recovered (persistent) amusia had greater GMV decrease (i.e., atrophy) in the right STG and MTG and white matter volume (WMV) decrease in the right MTG over a 6-month follow-up compared to non-amusic patients. Additionally, in a more recent paper, insular stroke lesions were associated with musical short-term memory deficits (Hirel et al., 2017).

In this study, we aim to extend and replicate the previous findings by utilizing a longitudinal design (acute and 6-month post-stroke) in a pooled cohort of altogether 90 stroke patients drawn from two independent cohorts (Helsinki, *N* = 47 and Turku, *N* = 43). Specifically, by applying VLSM on the pooled data and comparing non-amusic, recovered amusic, and non-recovered amusic patients, we sought to determine (i) which acute stage lesions would predict later recovery from amusia using VLSM with data from both cohorts (*N* = 90). Moreover, using longitudinal VBM analyses, we sought to determine (ii) whether the difference in GMV reported for non-recovered amusics vs. non-amusics in the Helsinki cohort (Sihvonen et al., 2016) would be replicated in the Turku cohort (*N* = 43) and (iii) whether the pooled data would show additional regions associated with amusia and also pinpoint regions specifically associated with amusia recovery. Furthermore, we aimed to (iv) provide more accurate outlook on gray and white matter changes associated with pitch and rhythm amusia. Based on our previous findings (Sihvonen et al., 2016), we hypothesized that lesions giving rise to acquired amusia would comprise at least the right basal ganglia, superior/middle temporal regions, and insula. We also hypothesized that in addition to the right temporal/subcortical areas also inferior frontal and parietal regions, especially in the right hemisphere, would be associated with amusia and its recovery. Furthermore, based on our previous results, we hypothesized that the right temporal GMV decreases would locate more posteriorly in pitch amusia and more anteriorly in rhythm amusia.

## Materials and methods

### Subjects and study design

Subjects (*N* = 100) were acute stroke patients enrolled in two music intervention studies in Helsinki and Turku, Finland. Fifty patients were recruited during 2004–2006 from the Department of Neurology, Helsinki University Central Hospital (HUCH) and 50 patients during 2013–2015 from the Department of Clinical Neurosciences, Turku University Hospital (Tyks). All patients had an MRI-verified acute ischemic stroke or intracerebral hemorrhage in the left (*N* = 49) or right (*N* = 51) hemisphere and subsequent cognitive and/or motor deficits, and they were all right-handed. Patients with hearing loss, prior neurological or psychiatric disease, or substance abuse were not included. All subjects gave written informed consent in accordance with the Declaration of Helsinki. The protocol was approved by the Ethics Committees of the HUCH and the Hospital District of Southwest Finland. All patients received standard medical treatment and rehabilitation for stroke. In both studies, all subjects underwent an MRI within 3 weeks of the stroke onset (acute stage) and at 6-month post-stroke stage. Behavioral assessment was performed at the acute and 3-month post-stroke stages. Out of the 100 recruited patients, 90 patients completed the 6-month MRI follow-up (Helsinki *N* = 47, Turku *N* = 43) and were included in the present study. The demographic and clinical characteristics of the patients are presented in Table 1.

**Table 1:** Demographic and clinical characteristics of the patients.

	**Helsinki and Turku patients (*****N*** = **90)**				**Turku patients (*****N*** = **43)**			
	**Non-recovered amusic (*n* = 37)**	**Recovered amusic (*n* = 16)**	**Non-amusic (*n* = 37)**	***p*-value**	**Non-recovered amusic (*n* = 18)**	**Recovered amusic (*n* = 6)**	**Non-amusic (*n* = 19)**	***p*-value**
**DEMOGRAPHIC VARIABLE**								
Gender (male/female)	20/17	9/7	20/17	0.987 (χ^2^)	12/6	4/2	8/11	0.273 (χ^2^)
Age (years)	61.1 (10.8)	55.9 (11.6)	55.7 (12.3)	0.096 (*F*)	59.7 (13.9)	53.2 (15.7)	54.9 (14.5)	0.491 (*F*)
Education (years)	10.7 (3.8)	12.4 (4.0)	14.4 (3.58)	0.000 (*F*)	12.1 (4.0)	13.4 (2.7)	15.6 (3.7)	0.024 (*F*)
**MUSIC BACKGROUND (PRE-STROKE)**								
Formal music training^a^	0.4 (1.3)	1.9 (2.1)	3.6 (1.4)	0.105 (*K*)	0.0 (0.0)	1.0 (1.7)	0.7 (1.6)	0.089 (*K*)
Instrument playing^a^	0.0 (0.0)	1.0 (1.6)	2.8 (1.7)	0.166 (*K*)	0.8 (1.8)	2.0 (2.4)	1.9 (2.2)	0.151 (*K*)
Music listening prior to stroke^b^	0.4 (1.1)	1.2 (1.8)	3.8 (1.4)	0.092 (*K*)	2.8 (1.7)	3.0 (1.8)	3.2 (1.7)	0.687 (*K*)
**CLINICAL VARIABLE**								
Aphasia (no/yes)^c^	20/17	8/8	20/17	0.957 (χ^2^)	6/12	2/4	9/10	0.646 (χ^2^)
BDAE-ASRS	4.3 (0.9)	4.3 (0.9)	4.4 (0.9)	0.859 (*K*)	4.2 (0.7)	4.2 (0.8)	4.4 (0.6)	0.494 (*K*)
MBEA total score %	54.8 (8.4)	68.4 (15.6)	84.4 (6.0)	0.000 (*F*)	54.4 (6.2)	68.9 (2.7)	83.9 (5.0)	0.000 (*K*)
Lesion laterality (left/right)	11/26	7/9	25/12	0.005 (χ^2^)	4/14	3/3	15/4	0.003 (χ^2^)
Lesion volume in cm^3^	69.3 (51.4)	48.2 (46.3)	31.1 (39.6)	0.000 (*F*)	78.6 (55.9)	35.3 (25.9)	36.5 (46.1)	0.023 (*K*)

*Data are mean (SD) unless otherwise stated. χ2, chi-square test; BDAE-ASRS, Boston Diagnostic Aphasia Examination—Aphasia Severity Rating Scale; K, Kruskal–Wallis test; MBEA, Montreal Battery of Evaluation of Amusia*.

a *Numbers denote values on a Likert scale where 0 = no, 1 = less than 1 year, 2 = 1–3 years, 3 = 4–6 years, 4 = 7–10 years, and 5 = more than 10 years of training/playing*.

b *Numbers denote values on a Likert scale with a range 0 (does never) to 5 (does daily)*.

c *Classification based on the Boston Diagnostic Aphasia Examination—Aphasia Severity Rating Scale*.

### Behavioral assessment

Following the methodology of our primary study (Sihvonen et al., 2016), music perception was evaluated using a shortened version (Särkämö et al., 2009) of the Montreal Battery of Evaluation of Amusia (MBEA; Peretz et al., 2003), the most widely used, gold standard method for diagnosing amusia. MBEA was assessed at the acute stage and at the 3-month post-stroke stage as a part of a larger neuropsychological testing battery. The average score of the Scale and Rhythm subtests of MBEA was utilized as an index of overall music perception (referred to hereafter as MBEA total score). Following the cut-off values of the original MBEA (Peretz et al., 2003) applied in our previous studies (Särkämö et al., 2009; Sihvonen et al., 2016), patients with the MBEA total score <75% were classified as amusic. Based on the MBEA at the 3-month stage, amusic patients were further divided to those who were tested non-amusic (recovered amusics) and those remaining amusic (non-recovered amusics) based on the MBEA cut-off value.

This classification yielded 19 non-amusics (NAs), 6 recovered amusics (RAs), and 18 non-recovered amusics (NRAs) in the Turku cohort and 37 NAs, 16 RAs, and 37 NRAs in the combined Helsinki-Turku cohort. To evaluate pitch and rhythm amusia separately, similar principle was applied to the Scale and Rhythm subtest scores. Patients with Scale subtest score <73% in the acute stage were defined as pitch-amusic [*N* = 50, non-pitch-amusic (pNA) *N* = 40]. At the 3-month stage, 17 patients were classified as recovered pitch-amusics (pRA) and 33 as non-recovered pitch-amusics (pNRA). Rhythm subtest was evaluated with cut-off score <77%: 28 non-rhythm-amusics (rNA), 37 non-recovered rhythm-amusics (rNRA), and 25 recovered rhythm-amusics (rRA). Overall, in both the Turku and the combined cohort, the three groups were relatively well-matched with respect to demographic and clinical variables (Table 1). Education years and acute stage lesion volume showed a group difference, and these variables were therefore included as covariates in the analyses. The RAs and NRAs showed a significant difference in acute stage MBEA total score both in the Turku cohort (*p* < 0.001) and the combined (*p* = 0.003) cohort, suggesting that poor recovery of amusia was linked to its initial severity. The patients in both cohorts were originally recruited to a music-based intervention study (for the results of Helsinki study, please see Särkämö et al., 2008, 2010a, 2014; the results of Turku study have not been published yet). To verify that the intervention did not have an effect on the amusia analyses, we calculated a mixed-model ANOVA with Time (Acute/3-month) and Group (3 intervention arms) using the pooled sample. No significant Time × Group interaction effect was found in the MBEA total score (*p* = 0.248), suggesting that the music intervention did not have any effect on the recovery of amusia and, therefore, does not impact the results of the present study.

### MRI data acquisition and preprocessing

Patients from the Helsinki study were scanned with a 1.5T Siemens Vision scanner (Siemens Medical Solutions, Erlangen, Germany) of the HUCH Department of Radiology to obtain high-resolution T1 images (flip angle = 15°, TR = 1,900 ms, TE = 3.68 ms, voxel size = 1.0 × 1.0 × 1.0 mm). Patients from the Turku study were scanned using a 3T Siemens Verio scanner (Siemens Medical Solutions, Erlangen, Germany) of the Medical Imaging Centre of Southwest Finland and T1-weighted MPRAGE were obtained (flip angle = 9°, TR = 2,300 ms, TE = 2.98 ms, voxel size = 1.0 × 1.0 × 1.0 mm).

**Figure 1:**
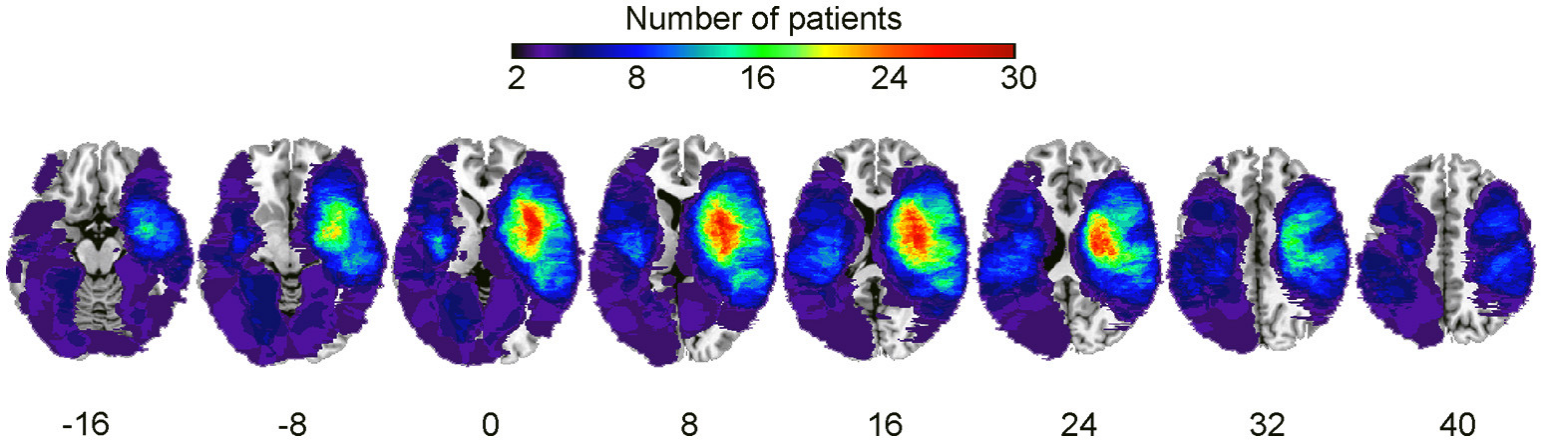
Lesion overlap map summing all subjects. Lesion distribution for the whole sample (*N* = 90). The warmer the areas, the greater the lesion overlap. Accordingly, the color scale ranges from 2 to 30 overlapping subjects.

Preprocessing steps equivalent to the primary study (Sihvonen et al., 2016) were carried out. First, to achieve optimal normalization of MRI images containing stroke lesions, cost function masking (CFM) was applied (Brett et al., 2001). Using CFM prevents post-registration lesion shrinkage and out-of-brain distortion (Ripollés et al., 2012). To define the CFMs, A.J.S. and T.S. created binary masks of the stroke lesions by manually depicting the precise lesion boundaries on a slice by slice basis using T1 images of individual patients. Lesion tracing was carried out by using MRIcron software package (http://people.cas.sc.edu/rorden/mricron/index.html; Rorden and Brett, 2000). A sum image of all patients' lesions (from both cohorts) is shown in Figure 1.

T1 images and the created lesion masks were processed using the Statistical Parametric Mapping software (SPM8, Wellcome Department of Cognitive Neurology, UCL) under MATLAB 8.4.0 (The MathWorks Inc., Natick, MA, USA, version R2014b). T1 images were segmented into gray matter (GM), white matter (WM), and cerebrospinal fluid probability maps using CFM and unified segmentation (Ashburner and Friston, 2005) with medium regularization. The created probability maps were then normalized into the MNI space (Montreal Neurological Institution). This technique corresponds to our primary study (Sihvonen et al., 2016) and has been widely used in stroke patients (Crinion et al., 2007; Andersen et al., 2010; Ripollés et al., 2012). The GM and WM images were modulated to preserve the original signal strength. Residual inter-individual variability was reduced by smoothing the GM and WM probability maps using an isotropic spatial filter (FWHM = 6 mm). Lastly, the binary lesion masks created in native space were also registered to MNI space.

### Voxel-based lesion-symptom mapping

Using the normalized acute stage lesion maps, VLSM was carried out with the Non-Parametric Mapping software (Chris Rorden's NPM, version 6 June 2013) in the combined Helsinki-Turku cohort (*N* = 90) adding 13 patients to the original VLSM study (Sihvonen et al., 2016) for more statistical power. Continuous VLSM analyses were carried out using the acute stage MBEA total score, Rhythm score, and Scale score. The following binary VLSM analyses were performed: NRA vs. NA, NRA vs. RA, and RA vs. NA. As the acute stage Rhythm and Scale subtest scores correlated strongly (*r* = 0.63), only the continuous analyses in rhythm or pitch amusia were carried out (note that the resulting maps show a great overlap and binary analyses would have yielded similar results). All voxels damaged at least in 10% of the patients were included in the statistical analysis (Dovern et al., 2011; Mirman et al., 2015; Timpert et al., 2015; Sihvonen et al., 2016). Multiple comparisons were accounted for with False Discovery Rate (FDR) correction with *p* < 0.05 threshold.

### Voxel-based morphometry

Voxel-based morphometric analysis was carried out using SPM8 software. Our original VBM study was based on the Helsinki cohort (*N* = 47), which is included in the present study. Therefore, in the current study with a higher statistical power, VBM was carried out using both the replication cohort (Turku; *N* = 43) and the combined Helsinki-Turku cohort (*N* = 90). The preprocessed GM and WM images were entered into a second-level analysis using a Group (NA/RA/NRA) × Time (Acute/6 months) mixed between-within subjects analysis of variance (ANOVA). Three different Group (NA > NRA, NA > RA, and RA > NRA) × Time (6 months > Acute) contrasts were calculated. Rhythm and pitch amusia were evaluated using preceding contrasts but with pNRA, pRA, and pNA, and rNRA, rRA, and rNA groups. Results were thresholded at a whole-brain uncorrected *p* < 0.005 at the voxel level (extent threshold: *k* > 100 voxels). Only clusters surviving an FWE-corrected *p* < 0.05 threshold are reported. Neuroanatomical areas were identified using the Automated Anatomical Labeling Atlas (Tzourio-Mazoyer et al., 2002) included in the xjView toolbox (http://www.alivelearn.net/xjview/). In addition to the previously determined covariates (education and lesion size), a covariate for the scanner was added in the gray and white matter VBM analyses, since the Turku and Helsinki patients were scanned with two different MRI scanners.

## Results

### Voxel-based lesion-symptom mapping: pooled cohort

#### Amusia

**Figure 2:**
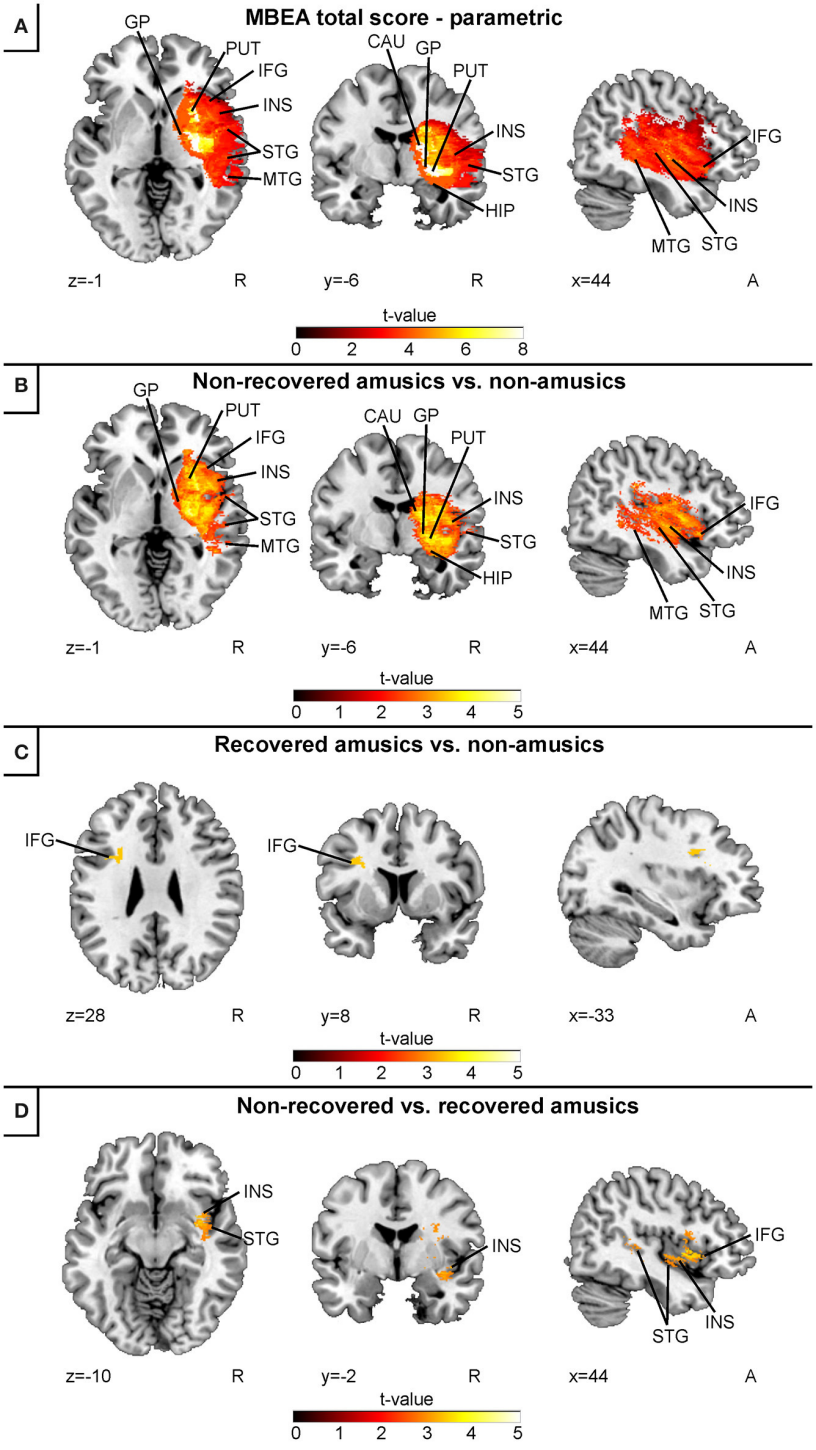
Acute stage VLSM results for amusia: pooled analysis. **(A)** MBEA total score continuous analysis; **(B)** comparison between non-recovered amusic vs. non-amusic patients; **(C)** comparison between recovered vs. non-amusic patients; and **(D)** comparison between non-recovered vs. recovered amusic patients. Neurological convention is used with MNI coordinates at the bottom left of each slice. All statistical maps are thresholded at an FDR-corrected *p* < 0.05 threshold, except for the panel **(D)**, which is thresholded at uncorrected *p* = 0.005 (*t* = 2.81). Critical brain structures are labeled. CAU, caudate; GP, globus pallidus; HIP, hippocampus; IFG, inferior frontal gyrus; INS, insula; MTG, middle temporal gyrus; PUT, putamen; STG, superior temporal gyrus.

In the continuous acute stage VLSM analysis of all subjects (combined Helsinki–Turku cohort), low acute stage MBEA total scores were associated with a lesion area comprising the right temporal (STG, MTG), and subcortical (caudate, putamen, globus pallidus) regions as well as the right IFG, hippocampus and insula (Figure 2A). In the binary acute stage VLSM analyses, a direct comparison between the non-recovered amusic (NRA) and non-amusic (NA) patients yielded essentially the same results (Figure 2B). In contrast to this extensive right hemispheric lesion pattern, a comparison between the recovered amusics (RAs) and NAs showed a smaller lesion localized at the left IFG (Figure 2C). Comparison between the NRAs and RAs did not yield any significant effects using the FDR-correction, but with slightly less stringed statistical criteria (*p* = 0.005 uncorrected), the NRAs showed a distinct lesion pattern in the right STG, IFG, and insula compared to the RAs (Figure 2D).

**Figure 3:**
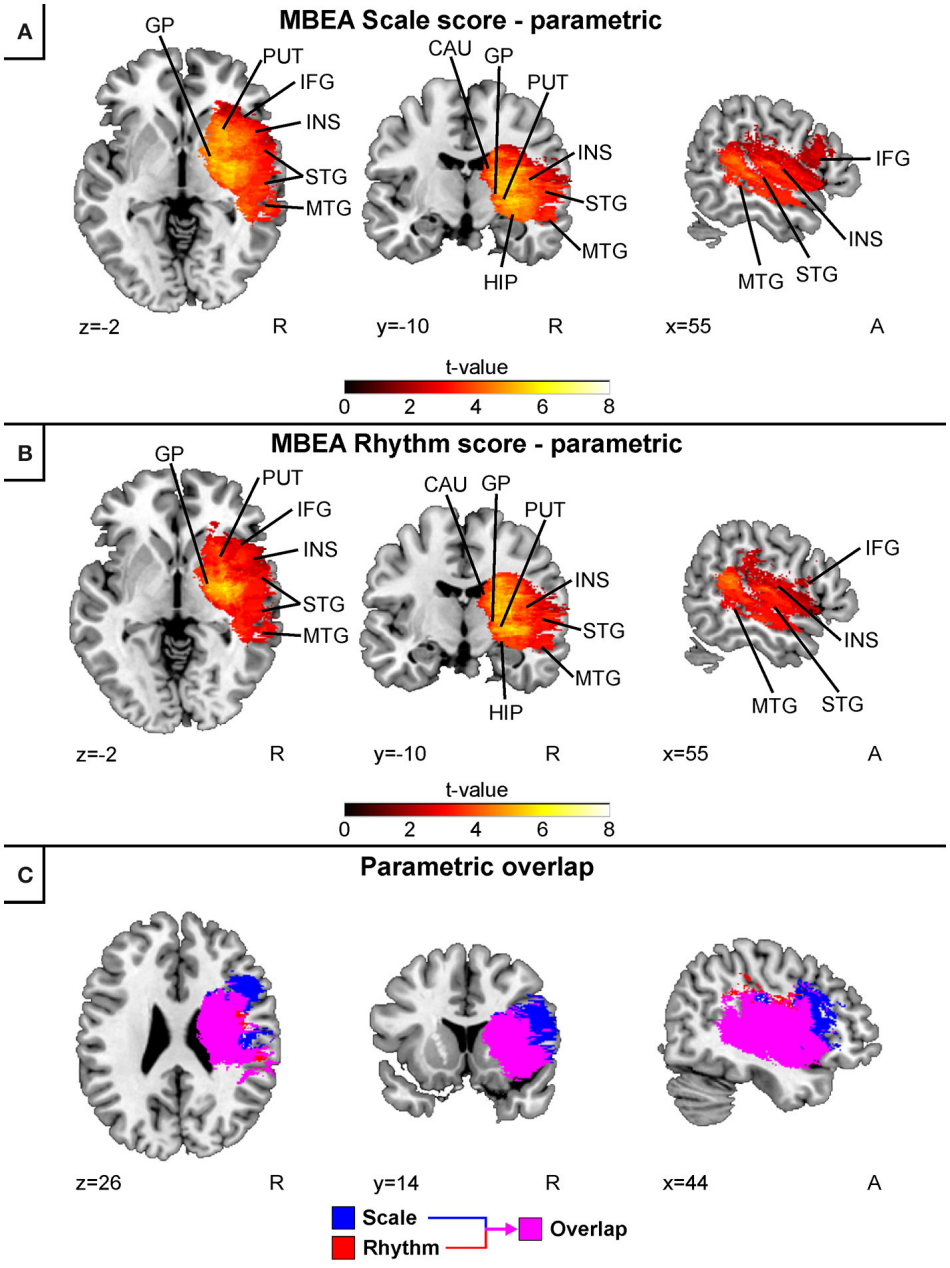
Acute stage VLSM results for scale and rhythm amusia: pooled analysis. Continuous analyses of **(A)** MBEA Scale score and **(B)** MBEA Rhythm score. **(C)** is an overlap image comparing Rhythm (red) and Scale (blue) subtests' continuous results. Neurological convention is used with MNI coordinates at the bottom left of each slice. All statistical maps are thresholded at an FDR-corrected *p* < 0.05 threshold. Critical brain structures are labeled. CAU, Caudate; GP, globus pallidus; HIP, hippocampus; IFG, inferior frontal gyrus; INS, insula; MTG, middle temporal gyrus; PUT, putamen; STG, superior temporal gyrus.

Separate continuous VLSM analyses for the acute stage MBEA Scale and Rhythm scores indicated largely overlapping results (Figure 3). In both analyses, lesion patterns comprised right temporal (STG, MTG) and subcortical (caudate, putamen, globus pallidus) as well as the right hippocampus, insula, and IFG. Overlaying both results revealed that the lesion pattern associated with pitch amusia extended more anterolaterally compared to the rhythm amusia lesion pattern (Figure 3C).

The present results extend our previously published results showing that—in addition to the right temporal, insular, and subcortical regions—stroke lesions associated with amusia comprise the right IFG and hippocampus. Additionally, in the current study with a higher statistical power, lesions comprising the left IFG were associated with less severe and transient amusia.

### Voxel-based morphometry: replication cohort

Longitudinal VBM analyses using the replication (Turku) cohort revealed similar results as our previously published study utilizing the Helsinki-cohort: Non-recovered amusia was associated with greater GMV decreases in the right STG and MTG, pitch amusia with GMV decreases in the right MTG, and rhythm amusia with anterior temporal GMV decreases. Additional GMV and WMV findings were observed and the results for the replication cohort are presented in the Supplementary Material.

### Voxel-based morphometry: pooled cohort

#### Gray and white matter volume: amusia

**Figure 4:**
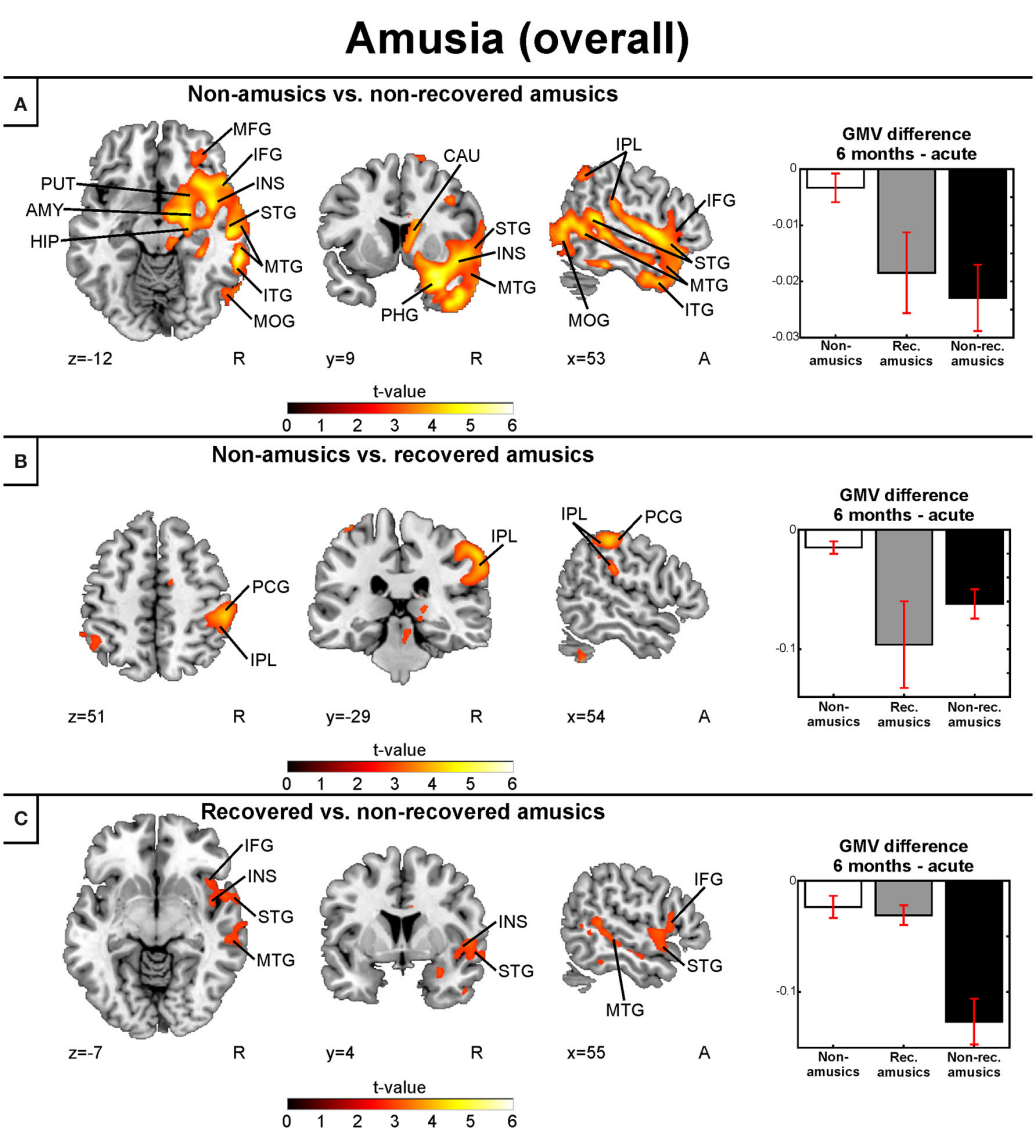
Gray matter VBM results of pooled analysis: amusia. Time (6 months > Acute) × Group interactions for gray matter volume. **(A)** Non-amusic vs. non-recovered amusics; **(B)** Non-amusic vs. recovered amusics; **(C)** Recovered vs. non-recovered amusics. Results are thresholded at a whole-brain uncorrected *p* < 0.005 at the voxel level (extent threshold: *k* > 100 voxels) using MNI coordinates. Only clusters surviving an FWE-corrected *p* < 0.05 threshold are reported and labeled (see also Table 2). Bar plots for GMV differences in 6 months—Acute in significant clusters (Table 2) are shown: bar = mean, error-bar = standard error of the mean. AMY, Amygdala; CAU, caudate; HIP, hippocampus; IFG, inferior frontal gyrus; INS, insula; IPL, inferior parietal lobule; ITG, inferior temporal gyrus; MFG, middle frontal gyrus; MOG, middle occipital gyrus; MTG, middle temporal gyrus; PCG, postcentral gyrus; PHG, parahippocampal gyrus; PUT, putamen; STG, superior temporal gyrus.

Similar to the replication cohort results, the pooled longitudinal VBM analysis yielded significant Time (6 months > Acute) × Group interactions for NA > NRA, NA > RA, and RA > NRA in GMV. However, these analyses extended our previous results by showing a set of new regions involved in amusia and its recovery. Compared to both NAs and RAs, the NRAs showed greater GMV decrease in the right temporal (STG, MTG) and frontal (IFG) regions. Compared to the NAs, additional GMV decrease in the NRAs was seen in other right temporal [Heschl's gyrus (HG)] and frontal [precentral gyrus (PreCG), middle frontal gyrus (MFG)] areas as well as in right parietal [inferior parietal lobule (IPL), superior parietal lobule (SPL)], subcortical (putamen, caudate, thalamus), and limbic [amygdala, hippocampus, parahippocampal gyrus (PHG)] areas as well as in the right insula (Table 2, Figures 4A,C). Furthermore, the RAs showed greater GMV decrease than the NAs in right parietal [IPL, postcentral gyrus (PCG)] areas (Table 2, Figure 4B).

**Table 2:** Gray matter volume decreases (6-month stage—acute stage) in amusia: pooled analysis.

**6 MONTHS** > **ACUTE**					
**Condition**	**Figure 4 panel**	**Area name**	**Coordinates**	**Cluster size**	***t*****-value**
Non-amusic > Non-recovered amusic	A	Right hippocampus	23 −13 −17	1,33,258	5.49^*^
		Right superior temporal gyrus (BA 22, 38)	54 3 −8		
		Right heschl's gyrus (BA 42)	64 −14 10		
		Right middle temporal gyrus (BA 19, 21, 22, 37, 39)	63 −38 −12		
		Right inferior temporal gyrus (BA 20, 21)	64 −14 −23		
		Right insula (BA 13)	44 −1 −4		
		Right precentral gyrus (BA 6)	54 −6 7		
		Right middle frontal gyrus (BA 10)	31 65 7		
		Right inferior frontal gyrus (BA 44)	55 13 9		
		Right superior parietal lobule (BA 7)	21 −73 55		
		Right inferior parietal lobule (BA 40)	46 −63 43		
		Right putamen	18 13 −7		
		Right caudate	9 12 9		
		Right thalamus	21 −28 14		
		Right amygdala	23 −1 −17		
		Right parahippocampal gyrus (BA 35)	31 −38 −14		
Non-amusic > Recovered amusic	B	Right inferior parietal lobule (BA 40)	60 −31 46	8,913	4.43^*^
		Right postcentral gyrus (BA 2)	64 −28 35		
Recovered amusic > Non-recovered amusic	C	Right inferior frontal gyrus (BA 47)	34 20 −16	11,739	3.33^*^
		Right superior Temporal Gyrus (BA 22, 38)	53 −43 10		
		Right middle Temporal Gyrus (BA 21)	63 −31 −18		
		Right inferior Temporal Gyrus (BA 20, 21)	61 −41 −21		

* *p < 0.005 FWE-corrected at the cluster level. BA, Brodmann area*.

**Figure 5:**
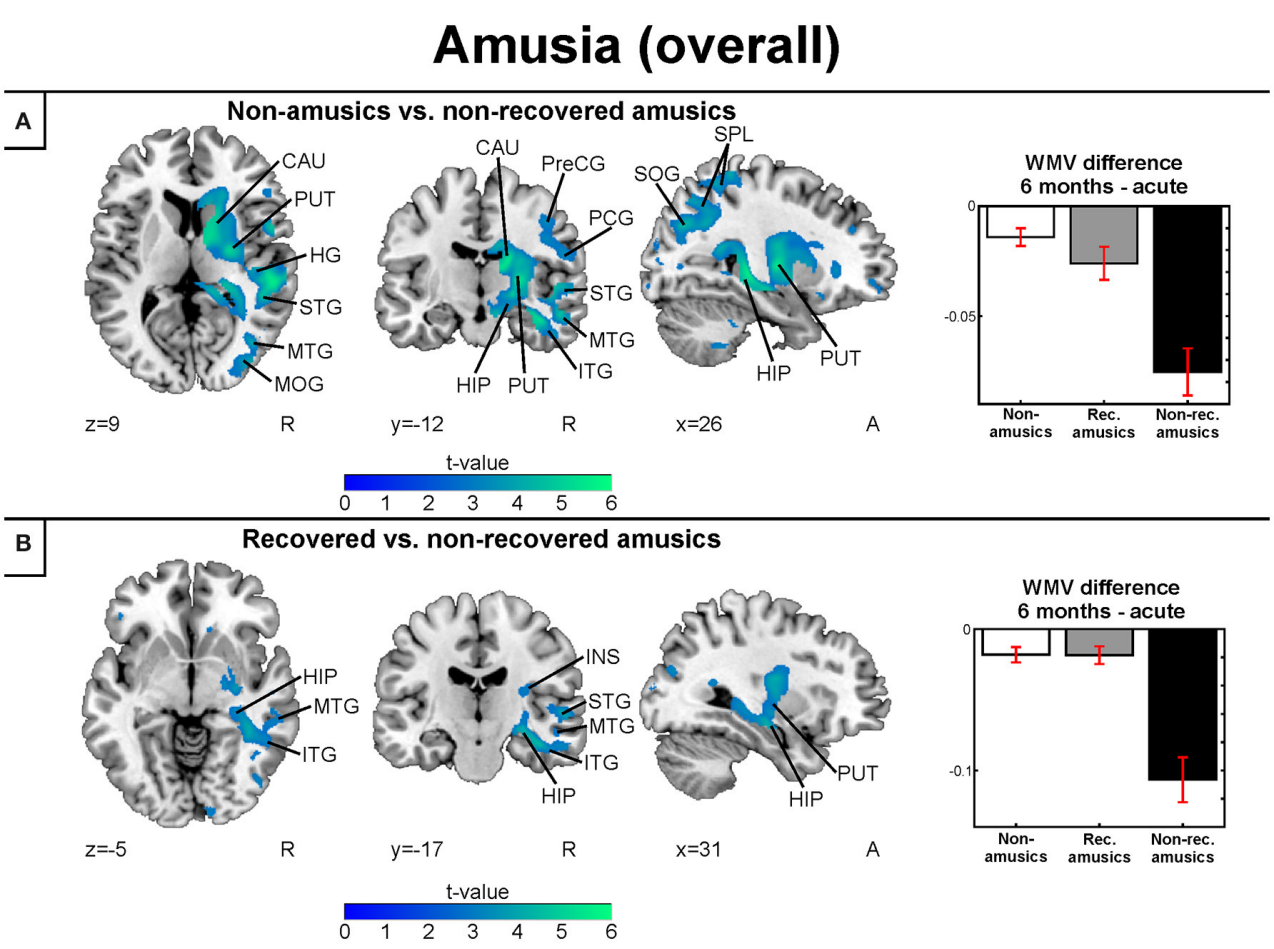
White matter VBM results of pooled analysis: amusia. Time (6 months > Acute) × Group interactions for white matter volume. **(A)** Non-amusic vs. non-recovered amusics; **(B)** Recovered vs. non-recovered amusics. Results are thresholded at a whole-brain uncorrected *p* < 0.005 at the voxel level (extent threshold: *k* > 100 voxels) using MNI coordinates. Only clusters surviving an FWE-corrected *p* < 0.05 threshold are reported and labeled (see also Table 3). Bar plots for GMV differences in 6 months—Acute in significant clusters (Table 3) are shown: bar = mean, error-bar = standard error of the mean. CAU, Caudate; HIP, hippocampus; HG, Heschl's gyrus; INS, insula; ITG, inferior temporal gyrus; MOG, middle occipital gyrus; MTG, middle temporal gyrus; PCG, postcentral gyrus; PUT, putamen; PreCG, precentral gyrus; SOG, superior occipital gyrus; SPL, superior parietal lobule; STG, superior temporal gyrus.

The longitudinal VBM analysis for WMV changes revealed significant Time (6 months > Acute) × Group interactions for NA > NRA and RA > NRA. Compared to both NAs and RAs, the NRAs showed greater WMV decrease in right temporal [MTG, inferior temporal gyrus (ITG)] and subcortical (putamen, caudate) areas as well as in the right hippocampus (Table 3, Figures 5A,B). Compared to the NAs, additional WMV decrease in the NRAs was observed in other right temporal (STG, HG), frontal (PreCG), parieto-occipital [IPL, SPL, PCG, superior occipital gyrus (SOG), middle occipital gyrus (MOG)], and insular areas (Table 3, Figure 5A).

**Table 3:** White matter volume decreases (6-month stage—acute stage) in amusia: pooled analysis.

**6 MONTHS** > **ACUTE**					
**Condition**	**Figure 6 panel**	**Area name**	**Coordinates**	**Cluster size**	***t-*****value**
Non-amusic > Non-recovered amusic	A	Right hippocampus	40 −23 −16	1,10,744	5.86^*^
		Right superior temporal gyrus	57 −31 17		
		Right heschl's gyrus	52 −26 9		
		Right middle temporal gyrus	55 −29 −6		
		Right inferior temporal Gyrus	55 −24 −23		
		Right insula	38 −24 20		
		Right superior parietal lobule	26 −55 44		
		Right inferior parietal lobule	38 −68 39		
		Right superior occipital gyrus	38 −77 24		
		Right middle occipital gyrus	38 −86 3		
		Right precentral gyrus	45 −12 41		
		Right postcentral gyrus	53 −12 22		
		Right putamen	26 −1 13		
		Right caudate	15 24 3		
Recovered > Non-recovered amusic	B	Right inferior temporal gyrus	42 −24 −17	29,103	4.48^*^
		Right middle temporal gyrus	58 −28 −10		
		Right hippocampus	35 −14 −16		
		Right putamen	26 −1 13		
		Right caudate	22 26 3		

* *p < 0.005 FWE-corrected at the cluster level*.

#### Gray and white matter volume: pitch amusia

**Figure 6:**
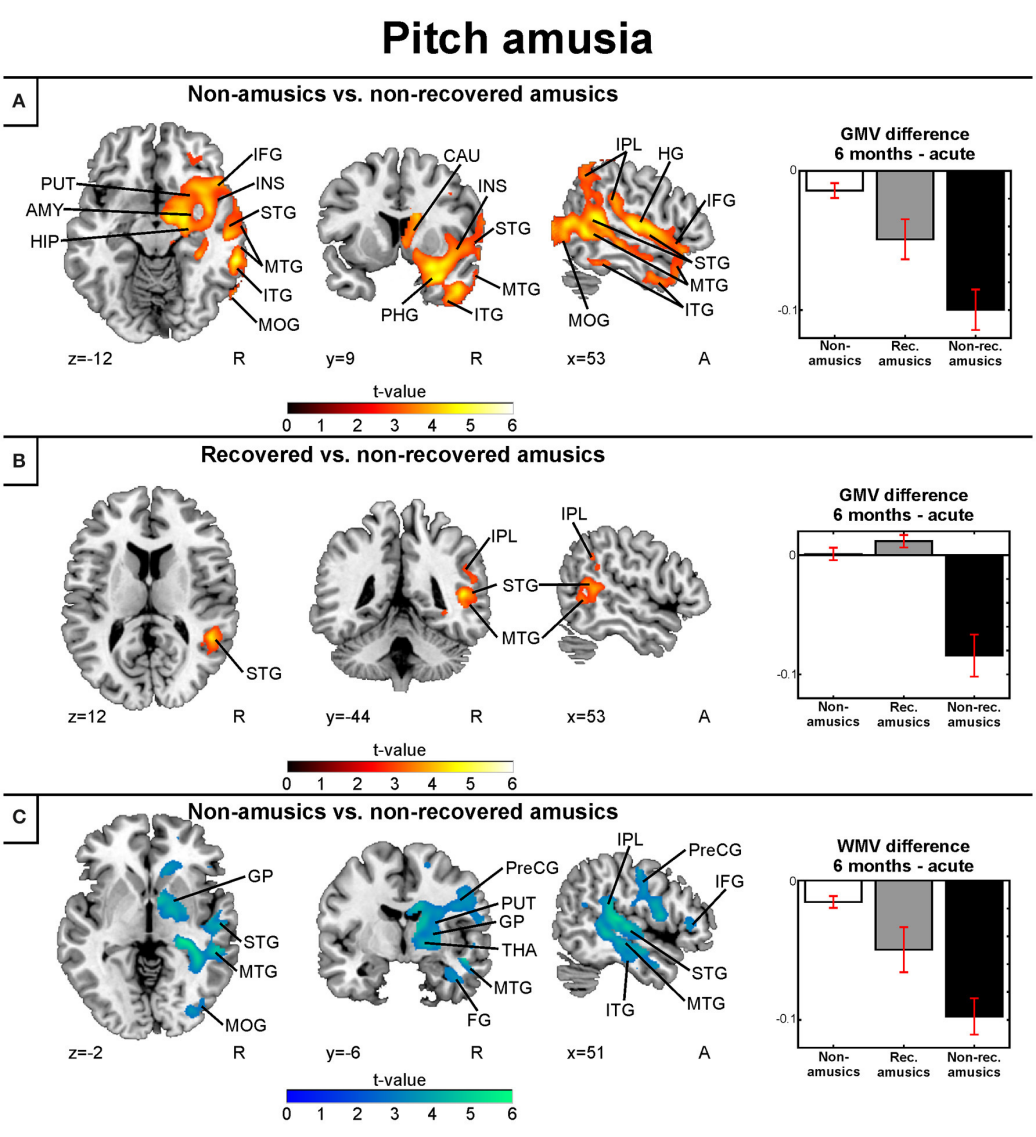
Gray and white matter VBM results of pooled analysis: pitch amusia. Time (6 months > Acute) × Group interactions for gray matter volume. **(A)** Non-amusic vs. non-recovered amusics; **(B)** Non-amusic vs. recovered amusics, and Time (6 months > Acute) × Group interactions for white matter volume **(C)** Non-amusic vs. non-recovered amusics. Results are thresholded at a whole-brain uncorrected *p* < 0.005 at the voxel level (extent threshold: *k* > 100 voxels) using MNI coordinates. Only clusters surviving an FWE-corrected *p* < 0.05 threshold are reported and labeled (see also Tables 4, 5). Bar plots for GMV differences in 6 months—Acute in significant clusters (Tables 4, 5) are shown: bar = mean, error-bar = standard error of the mean. AMY, amygdala; CAU, Caudate; FG, fusiform gyrus; GP, globus pallidus; HG, Heschl's gyrus; HIP, hippocampus; IFG, inferior frontal gyrus; INS, insula; IPL, inferior parietal lobule; ITG, inferior temporal gyrus; MFG, middle frontal gyrus; MOG, middle occipital gyrus; MTG, middle temporal gyrus; PCG, postcentral gyrus; PHG, parahippocampal gyrus; PreCG, precentral gyrus; PUT, putamen; STG, superior temporal gyrus; THA, thalamus.

Separate analysis of the MBEA Scale subtest yielded similar results as above: In the Scale subtest, the pNRAs showed greater GMV decrease in the right temporal (STG, MTG, ITG, HG), frontal (IFG), and parieto-occipital (IPL, MOG) regions as well as in the right insula and right subcortical (putamen, caudate, thalamus) and limbic structures (amygdala, hippocampus, PHG) compared to the pNA group (Table 4, Figure 6A). Compared to the pRAs, the pNRAs showed greater GMV decrease in the right STG, MTG, IPL (Table 4, Figure 6B).

**Table 4:** Gray matter volume decreases (6-month stage—acute stage) in pitch amusia: pooled analysis.

**6 MONTHS** > **ACUTE**					
**Condition**	**Figure 6 panel**	**Area name**	**Coordinates**	**Cluster size**	***t-*****value**
Non-amusic > Non-recovered amusic	A	Right middle temporal gyrus (BA 21, 39)	63 −38 −12	1,23,710	5.11^**^
		Right heschl's gyrus (BA 42)	43 −21 9		
		Right superior temporal gyrus (BA 22, 38, 39, 42)	61 −53 9		
		Right inferior temporal gyrus (BA 20)	61 −26 −23		
		Right insula (BA 13, 44)	43 −1 −5		
		Right inferior frontal gyrus (BA 44, 47)	61 17 14		
		Right inferior parietal lobule (BA 40)	46 −63 43		
		Right middle occipital gyrus (BA 37)	61 −65 −13		
		Right putamen	18 13 −7		
		Right caudate	9 12 9		
		Right thalamus	18 −30 13		
		Right amygdala	27 −7 −14		
		Right parahippocampal gyrus	22 −14 −25		
		Right hippocampus	16 −4 −14		
Recovered amusic > Non-recovered amusic	B	Right superior temporal gyrus (BA 22,39)	48 −45 10	3562	4.61^*^
		Right middle temporal gyrus (BA 21)	57 −47 4		
		Right inferior parietal lobule (BA 40)	51 −47 35		

* *p < 0.05 FWE-corrected at the cluster level*.

** *p < 0.005 FWE-corrected at the cluster level. BA, Brodmann area*.

Corresponding areas emerged in the longitudinal WMV analysis: Significant Time (6 months > Acute) × Group interaction was found for pNA > pNRA, showing greater WMV decrease for pNRA group in the right temporal (STG, MTG, ITG), frontal (IFG, PreCG), parieto-occipital (IPL, PCG, MOG), and subcortical regions (putamen, caudate, globus pallidus, thalamus; Table 5, Figure 6C).

**Table 5:** White matter volume decreases (6-month stage—acute stage) in pitch amusia: pooled analysis.

**6 MONTHS** > **ACUTE**					
**Condition**	**Figure 6 panel**	**Area name**	**Coordinates**	**Cluster size**	***t-*****value**
Non-amusic > Non-recovered amusic	C	Right middle temporal gyrus	59 −41 0	82,987	5.51^*^
		Right superior temporal gyrus	46 −22 4		
		Right inferior temporal gyrus	46 −22 −22		
		Right fusiform gyrus	43 −26 −15		
		Right inferior frontal gyrus	56 2 21		
		Right inferior parietal lobule	33 −50 45		
		Right middle occipital gyrus	33 −78 20		
		Right inferior occipital gyrus	33 −85 −2		
		Right precentral gyrus	56 −4 23		
		Right postcentral gyrus	56 −14 19		
		Right thalamus	17 −12 16		
		Right putamen	26 −1 13		
		Right globus pallidum	20 3 −2		
		Right Caudate	15 24 3		

* *p < 0.005 FWE-corrected at the cluster level*.

#### Gray and white matter volume: rhythm amusia

**Figure 7:**
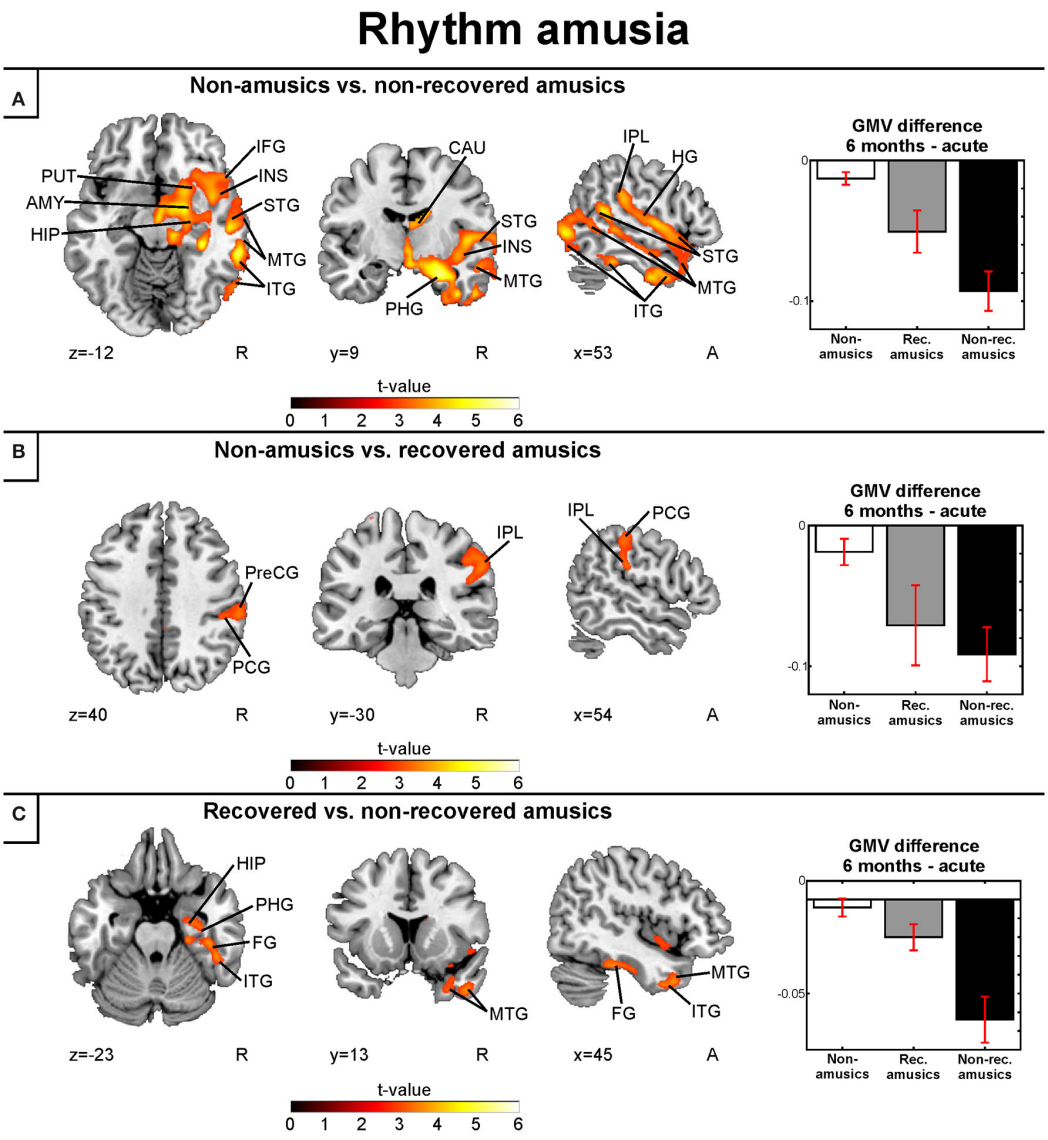
Gray matter VBM results of pooled analysis: rhythm amusia. Time (6 months > Acute) × Group interactions for gray matter volume. **(A)** Non-amusic vs. non-recovered amusics; **(B)** Non-amusic vs. recovered amusics; **(C)** Recovered vs. non-recovered amusics. Results are thresholded at a whole-brain uncorrected *p* < 0.005 at the voxel level (extent threshold: *k* > 100 voxels) using MNI coordinates. Only clusters surviving an FWE-corrected *p* < 0.05 threshold are reported and labeled (see also Table 6). Bar plots for GMV differences in 6 months—Acute in significant clusters (Table 6) are shown: bar = mean, error-bar = standard error of the mean. AMY, Amygdala; CAU, caudate; FG, fusiform gyrus; HG, Heschl's gyrus; HIP, hippocampus; IFG, inferior frontal gyrus; ITG, inferior temporal gyrus; INS, insula; IPL, inferior parietal lobule; ITG, inferior temporal gyrus; MTG, middle temporal gyrus; PCG, postcentral gyrus; PHG, parahippocampal gyrus; PreCG, precentral gyrus; PUT, putamen; STG, superior temporal gyrus.

When the MBEA Rhythm subtest was analyzed separately, we found that the rNRAs showed greater GMV decrease in the right temporal (STG, MTG, ITG, HG, fusiform gyrus), frontal (IFG), and parietal (IPL) regions as well as in the right insula and right subcortical (putamen, caudate, thalamus) and limbic structures (amydgala, hippocampus, PHG) compared to the rNA group (Table 6, Figure 7A). The rRA group showed also greater GMV decrease compared to the rNAs, but restricting to the right parieto-frontal area (IPL, PCG, and PreCG; Table 6, Figure 7B). Interestingly, the rNRAs showed greater GMV decrease in the right inferior temporal (ITG, fusiform gyrus) and limbic areas (hippocampus, PHG) compared to the rRAs (Table 6, Figure 7C).

**Table 6:** Gray matter volume decreases (6-month stage—acute stage) in rhythm amusia: pooled analysis.

**6 MONTHS** > **ACUTE**					
**Condition**	**Figure 7 panel**	**Area name**	**Coordinates**	**Cluster size**	***t-*****value**
Non-amusic > Non-recovered amusic	A	Right hippocampus (BA 28)	26 −14 −20	1,00,087	5.93^**^
		Right heschl's gyrus (BA 42)	47 −20 6		
		Right superior temporal gyrus (BA 13, 22, 38)	43 23 −31		
		Right middle temporal gyrus (BA 19, 21, 22, 37)	45 7 −40		
		Right inferior temporal gyrus (BA 20, 37)	54 −67 −13		
		Right insula (BA 22)	47 6 −6		
		Right inferior frontal gyrus (BA 47)	47 19 −8		
		Right inferior parietal lobule (BA 40)	62 −31 27		
		Right putamen	18 13 −7		
		Right caudate	9 12 9		
		Right thalamus	18 −30 13		
		Right amygdala	21 −7 −14		
		Right fusiform gyrus (BA 20, 36)	41 −18 −28		
		Right parahippocampal gyrus (BA 28)	22 −14 −25		
Non-amusic > Recovered amusic	B	Right inferior parietal lobule (BA 40)	59 −29 37	4,029	3.49^*^
		Right postcentral gyrus (BA 1, 2, 3)	59 −25 39		
		Right precentral gyrus (BA 4)	59 −23 44		
Recovered amusic > Non-recovered amusic	C	Right parahippocampal gyrus (BA 19, 35)	36 −33 −11	5,488	4.35^**^
		Right hippocampus (BA 28)	22 −12 −20		
		Right fusiform gyrus (BA 20, 36, 37)	37 −44 −5		
		Right middle temporal gyrus (BA 37)	41 13 −39		
		Right inferior temporal gyrus (BA 20, 37)	51 −40 −23		

* *p < 0.05 FWE-corrected at the cluster level*.

** *p < 0.005 FWE-corrected at the cluster level. BA, Brodmann area*.

**Figure 8:**
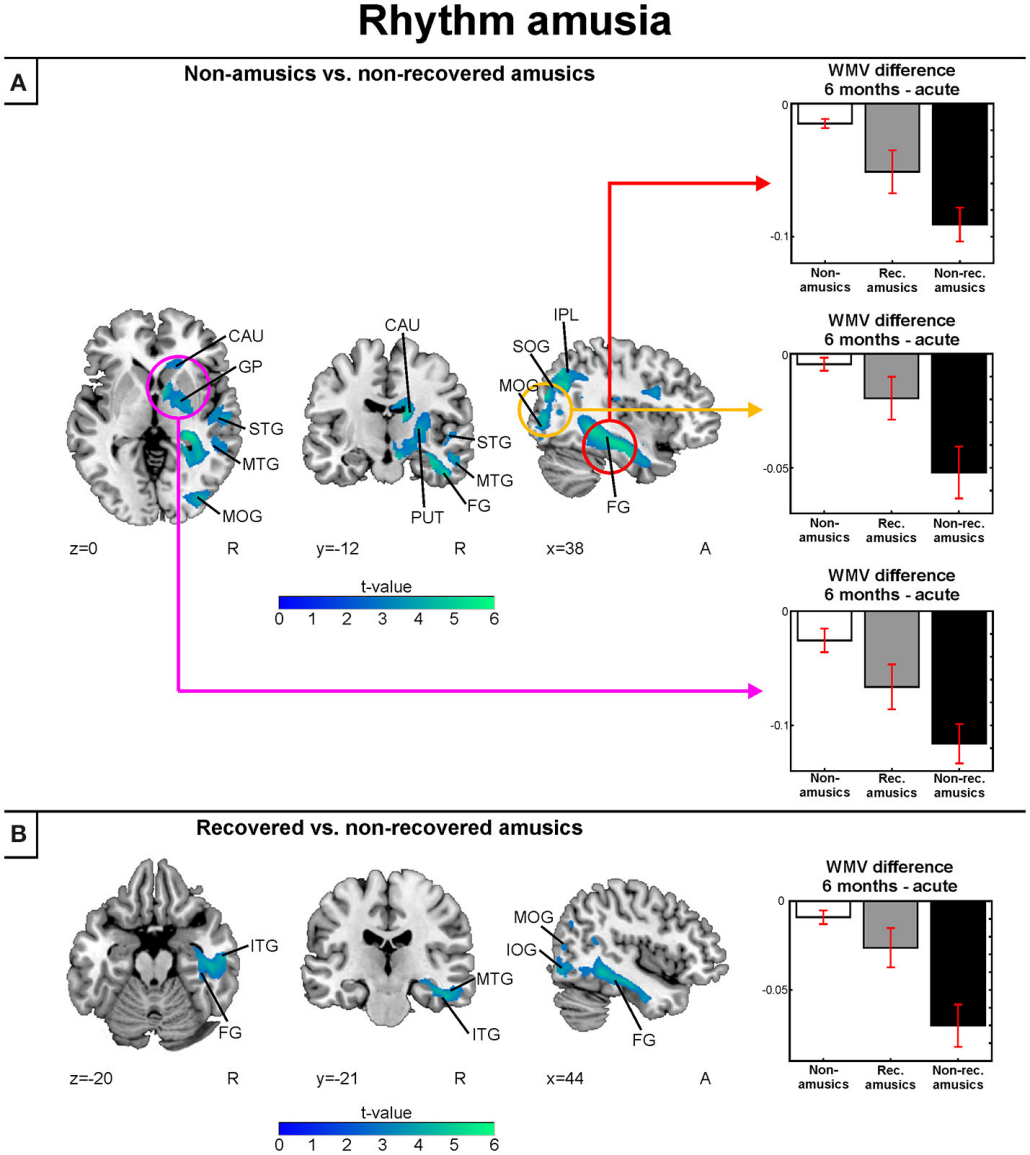
White matter VBM results of pooled analysis: rhythm amusia. Time (6 months > Acute) × Group interactions for white matter volume. **(A)** Non-amusic vs. non-recovered amusics; **(B)** Recovered vs. non-recovered amusics. Results are thresholded at a whole-brain uncorrected *p* < 0.005 at the voxel level (extent threshold: *k* > 100 voxels) using MNI coordinates. Only clusters surviving an FWE-corrected *p* < 0.05 threshold are reported and labeled (see also Table 7). Bar plots for GMV differences in 6 months—Acute in significant clusters (Table 7) are shown: bar = mean, error-bar = standard error of the mean. CAU, Caudate; FG, fusiform gyrus; GP, globus pallidus; IPL, inferior parietal lobule; ITG, inferior temporal gyrus; MOG, middle occipital gyrus; MTG, middle temporal gyrus; PUT, putamen; SOG, superior occipital gyrus; STG, superior temporal gyrus.

In the longitudinal WMV analysis, significant Time (6 months > Acute) × Group interaction were found for rNA > rNRA and rRA > rNRA. Compared to both the rNAs and rRAs, the rNRAs showed greater WMV decrease in the right inferior temporal (ITG, fusiform gyrus) and occipital areas (SOG, MOG, IOG; Table 7, Figures 8A,B). Additionally, compared to the rNAs, the rNRAs showed WMV decreases in the right STG, fronto-parietal regions (IFG, PreCG, PCG, IPL, precuneus), and PHG (Table 7, Figure 8A).

**Table 7:** White matter volume decreases (6-month stage—acute stage) in rhythm amusia: pooled analysis.

**6 MONTHS** > **ACUTE**					
**Condition**	**Figure 8 panel**	**Area name**	**Coordinates**	**Cluster size**	***t-*****value**
Non-amusic > Non-recovered amusic	A	Right fusiform gyrus	43 −25 −17	68,278	5.65^**^
		Right superior temporal gyrus	56 −28 9		
		Right middle temporal gyrus	57 −45 5		
		Right inferior temporal gyrus	47 −11 −31		
		Right parahippocampal gyrus	34 −2 −25		
		Right inferior parietal lobule	57 −40 22		
		Right precuneus	35 −69 41	12,533	5.43^**^
		Right middle occipital gyrus	35 −67 33		
		Right inferior occipital gyrus	31 −84 −5		
		Right precentral gyrus	55 5 11	3,252	3.81^*^
		Right inferior frontal gyrus	52 4 19		
		Right postcentral gyrus	60 −5 19		
		Right globus pallidum	23 −3 0		
		Right putamen	28 −12 4		
		Right caudate	19 27 0		
Recovered > Non-recovered amusic	B	Right middle occipital gyrus	39 −80 23	24,150	4.68^**^
		Right inferior occipital gyrus	34 −85 −2		
		Right middle temporal gyrus	54 −29 −12		
		Right inferior temporal gyrus	44 −44 −9		
		Right fusiform gyrus	32 −50 −8		

* *p < 0.05 FWE-corrected at the cluster level*.

** *p < 0.005 FWE-corrected at the cluster level*.

Compared to pitch amusia, the GMV change associated with rhythm amusia recovery was more anterior, locating in the anterior MTG and ITG. In contrast, recovered pitch amusia was associated with posterior superior temporal and inferior parietal GMV changes. Overall, rhythm amusia was associated with GMV decreases in right inferior temporal regions. In contrast, GMV decreases comprising the right STG/MTG and IPL were associated with pitch amusia. Specific WMV changes in rhythm amusia located in right inferior temporal occipital regions whereas in pitch amusia specific WMV changes were observed in the right MTG.

## Discussion

The aim of the present study to extend and replicate our previous VLSM and VBM results (Sihvonen et al., 2016) and, using a larger pooled sample of stroke patients and therefore having a higher statistical power, to provide a more precise and detailed account of the neural basis of acquired amusia and its recovery after stroke. Using both the replication cohort (*N* = 43; see Supplementary Material) and the larger pooled sample (*N* = 90) of stroke patients, we were able to ascertain our previous results (Sihvonen et al., 2016) that (i) acquired amusia was associated with an acute stage lesion pattern in right temporal, insular, and striatal areas and that (ii) non-recovered amusia was linked to longitudinal GMV decrease in right temporal areas, located posteriorly for pitch amusia and more anteriorly for rhythm amusia. Importantly, compared to the previous study, the larger pooled sample also enabled us to carry out direct lesion comparisons to evaluate lesion patterns associated with amusia recovery. Furthermore, using the larger pooled sample yielded additional areas related to amusia, both in VLSM and VBM, providing a more comprehensive picture of the lesions and longitudinal structural changes associated with different recovery trajectories of acquired amusia. The main novel finding was that (i) more severe and persistent amusia was associated with an extensive pattern of acute stage lesions and longitudinal GMV/WMV changes in the right hemisphere, which included not only temporal, insular, and striatal areas but also frontal, parietal, and limbic areas, and, conversely, (ii) less severe and transient amusia was linked to lesions specifically in left frontal areas as well as GMV changes in right parietal areas, and (iii) compared to the non-recovered amusia, recovered amusia was related to less GMV decrease in the temporal lobe, located more posterosuperiorly in pitch amusia and more inferoanteriorly in rhythm amusia.

### Temporal areas

Across the VLSM and VBM analyses, several lateral and medial temporal regions in the right hemisphere were found to be related to amusia and its recovery. In the acute stage VLSM, converging results from both continuous analyses of MBEA total score, Scale and Rhythm subtest scores and binary analyses comparing NRAs and NAs showed that severe non-recovered amusia was caused by lesions in the right STG, MTG, and insula. This is well in line with findings from neuroimaging studies of healthy subjects implicating superior temporal (Griffiths et al., 1998; Gutschalk et al., 2002; Patterson et al., 2002; Tramo et al., 2002; Hyde et al., 2008; Kumar et al., 2016) regions in pitch and/or melodic processing. Similarly, studies of both acquired amusia (Liegeois-Chauvel et al., 1998; Ayotte et al., 2000; Kohlmetz et al., 2003; Terao et al., 2006; Särkämö et al., 2010b; Hochman and Abrams, 2014; Sihvonen et al., 2016) and congenital amusia (Hyde et al., 2007; Albouy et al., 2013) have reported lesions/structural gray matter abnormalities specifically in right superior temporal and/or insular areas. Importantly, our novel VLSM and VBM results showed that the right STG/MTG has a crucial role also in amusia recovery as the NRAs had more lesions at the acute stage and also more GMV and WMV decrease from acute to 6-month stage compared to the RAs, suggesting that initial damage and further atrophy of these regions is a strong indicator for severe amusia that has a poor prognosis.

Separate VBM analyses for pitch and rhythm amusia showed that recovered pitch amusia was related to smaller GMV decrease in the right posterior STG/MTG compared to non-recovered pitch amusia. In contrast, recovered rhythm-amusics showed less GMV decrease in the anterior MTG, ITG, and fusiform gyrus as well as less WMV in the ITG and fusiform gyrus than the rNRAs. These results provide crucial information for pinpointing the functional organization of the right temporal lobe for pitch and rhythm processing, and moreover, they are in line with previous observations where anterior temporal lesions have been associated with rhythm processing deficits (Kester et al., 1991; Liegeois-Chauvel et al., 1998) and posterior temporal structures to spectral processing (Warren et al., 2005; Jamison et al., 2006; for a meta-analysis, see Samson et al., 2011).

### Frontal and parietal regions

Compared to our previous results, in addition to temporal areas, frontal, and parietal regions showed an association with amusia in the present study with higher statistical power. In the VLSM and VBM analyses, the NRAs had more lesions and more GMV decrease in the right IFG compared to both the NAs and the RAs. This pattern in NRAs extended also to the right MFG and PreCG when compared to the NAs. These results suggest that, in addition to the right STG/MTG, the right IFG-MFG-PreCG appears to be another crucial hub in acquired amusia as its lesions and atrophy are linked to initial severity and poor recovery. Previously, these areas have been implicated in the sequencing of auditory information and structural (syntactic) analysis of music (Koelsch, 2005; Tillmann et al., 2006; Bianco et al., 2016) in healthy subjects.

Reduced activation and connectivity and gray and white matter abnormalities in the right IFG have also been reported in congenital amusia (Hyde et al., 2006, 2007, 2011; Albouy et al., 2013). Interestingly, the left IFG emerged as the only lesion site in the VLSM contrast between the RAs and NAs. Along with its right hemisphere homolog, the left IFG (or Broca's area) has been found to process syntactic information in both language and music (Maess et al., 2001; Kunert et al., 2015) and, conversely, that its damage impairs both linguistic and musical syntactic processing (Patel et al., 2008; Sammler et al., 2011). Our results provide support for the role of the left IFG in amusia, but suggest that its acute damage results in less severe and transient form of amusia (Kumar et al., 2016). VBM results also showed that compared to the NAs, both the NRAs and the RAs had more GMV/WMV decrease also in right parietal areas, especially in right IPL/SPL, suggesting that this region is generally associated with amusia, regardless of its recovery. Right parietal areas have been implicated in neuroimaging studies to be involved in the processing of more higher-level melodic features, such as tonality (Foster et al., 2013; Royal et al., 2016), as well as in tonal working memory and recognition (Jerde et al., 2011; Schulze et al., 2011; Albouy et al., 2017). Importantly, in the pitch domain, recovered pitch amusics showed more GMV in the right IPL than the pNRAs.

### Subcortical and limbic regions

Finally, the pattern of acute lesions and the longitudinal GMV/WMV decreases observed for NRAs vs. NAs also encompassed right subcortical areas, both in the striatum (caudate, putamen) and in limbic areas (amygdala, hippocampus, parahippocampal gyrus). Together with the GMV decrease in PreCG, the striatal changes are most likely related to rhythmic deficits, as these regions have been strongly linked to rhythm processing in neuroimaging studies (Penhune et al., 1998; Grahn and Brett, 2009; Grahn and Rowe, 2009; Alluri et al., 2012). In line with the key role of rhythm in mediating the emotional valence and arousal induced by music, musical pulse, or rhythm has also been shown to engage amygdala and hippocampus (Alluri et al., 2012; Toiviainen et al., 2014), the latter playing a role also in auditory working memory (Burunat et al., 2014). Musical training has also been linked to increased hippocampal and amygdala volume (Oechslin et al., 2013; Dohn et al., 2015; Vaquero et al., 2016), providing converging support for our finding of decreased volume of these structures in non-recovered amusia.

Converging evidence from neuroimaging studies in healthy subjects utilizing natural music stimuli suggest that music perception and analysis is a highly wide-spread process in the brain, engaging a large-scale network of bilateral temporal, frontal, parietal, and subcortical regions (Schmithorst, 2005; Brattico et al., 2011; Alluri et al., 2012; Burunat et al., 2014; Toiviainen et al., 2014). In contrast, structural deficits, indicated by volumetric and cortical thickness measures that have been reported in congenital amusia have thus far been limited to right superior temporal and inferior frontal areas (Hyde et al., 2006, 2007; Albouy et al., 2013). Our results support the strong right hemispheric basis for amusia, but suggest that in acquired amusia the lesion and atrophy pattern underlying the severity and persistence of the deficit might be more extensive and wide-spread. While the statistical power in the VLSM analyses in the current study was higher in the right hemisphere than in the left hemisphere, the observed longitudinal results do not appear to be due to a simple mass effect since the overall volume of the lesions was controlled for in the VBM analyses. However, future studies investigating acquired amusia in patients with left hemisphere damage would be of great interest. As stroke may have impact on other modalities, the effect of other cognitive deficits on MBEA performance should be also taken into consideration.

On the other hand, given that the large-scale atrophy pattern extended from right prefrontal (IFG/MFG) all the way to right posterior (parietal/occipital) regions, which are not considered to be part of the music perception network, it is possible that this may reflect damage to the long-range white matter pathways, such as the inferior fronto-occipital fasciculus (IFOF). The IFOF is a ventral pathway that runs from its posterior terminations (inferior and middle occipital gyri, parietal lobe) through the external capsule, between the insula and putamen, and connects to multiple temporal (STG/MTG) and frontal (IFG/MFG) areas (Catani et al., 2002; Hau et al., 2016), thereby covering most of the lesion/atrophy areas observed in our NRA patients. Thus, far using diffusion weighted MRI (DW-MRI), amusia has been linked only to the right arcuate fasciculus (Loui et al., 2009), a dorsal pathway connecting the IFG and STG. However, this finding has been recently challenged using a larger sample of congenital amusics and multiple algorithms (Chen et al., 2015) and the crucial white matter connections affected in congenital amusia remain unclear (for a review, see Peretz, 2016).

In future, more research on the roles of the ventral and dorsal pathways in amusia is clearly needed. Furthermore, although the data presented here sheds more light on the recovery mechanisms in acquired amusia, an interesting idea would be to use machine learning methods to predict, by means of lesion data, not only which patients will become amusics, but also which one will recover (Rondina et al., 2016). Further research is needed, especially studies using multimodal MRI data.

## Author contributions

AR, SS, and TS designed research; AS and TS performed research; AS, PR, and TS analyzed data; AS, TS, PR, AR, and SS wrote the paper.

### Conflict of interest statement

The authors declare that the research was conducted in the absence of any commercial or financial relationships that could be construed as a potential conflict of interest.
