# Supplementary material for: Revisiting the Neural Basis of Acquired Amusia: Lesion Patterns and Structural Changes Underlying Amusia Recovery
Source: Front Neurosci. 2017 Jul 25;11:426. doi: 10.3389/fnins.2017.00426 (PMC5524924; doi:10.3389/fnins.2017.00426)

Supplementary Material

# Revisiting the neural basis of acquired amusia: Lesion patterns and structural changes underlying amusia recovery

Aleksi J. Sihvonen^1,2^, Pablo Ripollés^3,4,5^, Antoni Rodríguez-Fornells^3,4,6^, Seppo Soinila^7^, and Teppo Särkämö^2^.

^1^Faculty of Medicine, University of Turku, 20520 Turku, Finland
^2^Cognitive Brain Research Unit, Department of Psychology and Logopedics, Faculty of Medicine, University of Helsinki, 00014 Helsinki, Finland
^3^Cognition and Brain Plasticity Group, Bellvitge Biomedical Research Institute (IDIBELL), L’Hospitalet de Llobregat, 08907 Barcelona, Spain
^4^Department of Cognition, Development and Education Psychology, University of Barcelona, 08035 Barcelona, Spain
^5^Poeppel Lab, Department of Psychology, New York University, 10003 NY, USA
^6^Catalan Institution for Research and Advanced Studies, ICREA, Barcelona, Spain
^7^Division of Clinical Neurosciences, Turku University Hospital and Department of Neurology, University of Turku, 20521, Turku, Finland.

*Correspondence: Dr. Aleksi J. Sihvonen: ajsihv@utu.fi

**1. Results**

**1.1 Voxel-based morphometry: replication cohort**

**1.1.1 Grey and white matter volume: amusia**

Using longitudinal VBM, we first evaluated GMV changes in the replication cohort from Turku. There were significant Time (6 months > Acute) interactions for following group contrasts: NA > NRA, RA > NRA, and NA > RA. The NRA group showed greater GMV decrease in right temporal (STG), frontal [middle frontal gyrus (MFG), IFG], subcortical (putamen), and limbic (amygdala, hippocampus) structures as well as in the right insula than either the NA or the RA groups (Supplementary Table 1, Supplementary Figure 1A and 1C). Additionally, compared to the NAs, the NRAs had greater GMV decrease in the right temporal [MTG, Heschl’s gyrus (HG), inferior temporal gyrus (ITG)], frontal [precentral gyrus (PreCG)], and subcortical (caudate, and thalamus) regions as well as in limbic areas [parahippocampal gyrus (PHG)]. The RA group had greater GMV decrease in the right parietal lobe [inferior parietal lobule (IPL), postcentral gyrus (PCG)] than the NA group, as shown by a Time (6 months > Acute) x Group (NA > RA) interaction (Supplementary Table 1, Supplementary Figure 1B).

A longitudinal VBM analysis for WMV changes yielded significant Time (6 months > Acute) x Group interactions for NA > NRA and NA > RA. The NRAs had greater WMV decrease in the right temporal (STG, MTG, ITG), frontal (PreCG, PCG, IFG), subcortical (putamen, caudate, thalamus) regions as well as in the right insula and hippocampus than the NAs (Supplementary Table 2, Supplementary Figure 2A). The RAs showed greater WMV decrease in the right frontoparietal (IPL, PreCG, and PCG) areas than the NAs (Supplementary Table 2, Supplementary Figure 2B).

**1.1.2 Grey and white matter volume: pitch amusia**

In the Scale subtest, compared to the NAs, the NRAs showed greater GMV decrease in right temporal (STG, HG), frontal [MFG, IFG, PreCG], subcortical (putamen, caudate), and limbic (amygdala, hippocampus) regions as well as in the right insula (Supplementary Table 3, Supplementary Figure 3A). In contrast, the RAs showed greater GMV decrease in the right thalamus, caudate, and IPL compared to the NAs (Supplementary Table 3, Supplementary Figure 3B).

Longitudinally, compared to the NAs, the NRAs showed greater WMV decrease in right temporal (STG, MTG, ITG), frontal [MFG, PreCG], subcortical (putamen, caudate, GP), and limbic (thalamus, hippocampus, PHG) regions as well as in the right insula and PCG and the midbrain (Supplementary Table 4, Supplementary Figure 4A).

**1.1.3 Grey and white matter volume: rhythm amusia**

In the Rhythm subtest, the NRAs showed greater GMV decrease in right temporal (STG, MTG, fusiform gyrus), frontal [IFG], subcortical (caudate), and limbic (amygdala, hippocampus, PHG) regions compared to the NAs (Supplementary Table 5, Supplementary Figure 5A). In contrast, compared to the NA group, the RAs showed greater GMV decrease in the right PCG, left precuneus, and bilaterally in the IPL (Supplementary Table 5, Supplementary Figure 5B).

Significant Time (6 months > Acute) x Group interactions in WMV were found in NA > NRA and NA > RA. Compared to the NAs, the NRAs showed greater WMV decrease in the midbrain and bilaterally in the cerebellum as well as in right inferior temporal (ITG, fusiform gyrus), subcortical (caudate), and limbic (thalamus, hippocampus, PHG) regions (Supplementary Table 6, Supplementary Figure 6A). The RAs showed greater WMW decrease in the right PCG and paracentral lobule compared to the NAs (Supplementary Table 6, Supplementary Figure 6B).

**2. Tables and Figures**

Supplementary Table 1 Grey matter volume decreases (6-month stage – acute stage) in amusia: Turku cohort.

| **6 MONTHS > ACUTE** | | | | | |
| --- | --- | --- | --- | --- | --- |
| **Condition** | **Supplementary Figure 1 panel** | **Area name** | **Coordinates** | **Cluster size** | **t-value** |
| **Non-amusic > Non-recovered amusic** | **A** | Right Amygdala | 25 -1 -20 | 68942 | 5.26** |
|  |  | Right Inferior Frontal Gyrus (BA 13, 45, 47) | 37 20 -12 |  |  |
|  |  | Right Middle Frontal Gyrus (BA 9, 10) | 42 13 36 |  |  |
|  |  | Right Superior Temporal Gyrus (BA 22, 38, 42) | 38 21 -32 |  |  |
|  |  | Right Heschl’s Gyrus (BA 41) | 53 -19 12 |  |  |
|  |  | Right Middle Temporal Gyrus (BA 21, 38) | 47 10 -33 |  |  |
|  |  | Right Inferior Temporal Gyrus (BA 20) | 51 -2 -33 |  |  |
|  |  | Right Insula (BA 13) | 41 11 -7 |  |  |
|  |  | Right Precentral Gyrus (BA 43) | 62 -8 12 |  |  |
|  |  | Right Parahippocampal Gyrus (BA 35) | 21 -21 -18 |  |  |
|  |  | Right Putamen | 17 10 -7 |  |  |
|  |  | Right Hippocampus | 20 -12 -18 |  |  |
|  |  | Right Caudate | 14 9 16 | 8852 | 5.26** |
|  |  | Right Thalamus | 5 -10 15 |  |  |
| **Non-amusic > Recovered amusic** | **B** | Right Inferior Parietal Lobule (BA 40) | 60 -33 46 | 6249 | 5.22** |
|  |  | Right Postcentral Gyrus (BA 2) | 65 -28 35 |  |  |
| **Recovered amusic > Non-recovered amusic** | **C** | Right Amygdala | 23 0 -24 | 3768 | 4.23* |
|  |  | Right Hippocampus | 27 -8 -22 |  |  |
|  |  | Right Putamen | 16 10 -6 |  |  |
|  |  | Right Superior Temporal Gyrus (BA 38) | 37 18 -28 |  |  |
|  |  | Right Inferior Frontal Gyrus (BA 44, 45, 47) | 52 15 18 | 9344 | 3.70** |
|  |  | Right Middle Frontal Gyrus (BA 9) | 47 20 38 |  |  |
|  |  | Right Insula (BA 13) | 46 11 5 |  |  |
|  |  | Right Superior Temporal Gyrus (BA 22) | 54 1 4 |  |  |
| *p < 0.05 FWE-corrected at the cluster level | | | | | |
| **p < 0.005 FWE-corrected at the cluster level | | | | | |
| BA = Brodmann area | | | | | |

Supplementary Table 2 White matter volume decreases (6-month stage – acute stage) in amusia: Turku cohort.

| **6 MONTHS > ACUTE** | | | | | |
| --- | --- | --- | --- | --- | --- |
| **Condition** | **Supplementary Figure 2 panel** | **Area name** | **Coordinates** | **Cluster size** | **t-value** |
| **Non-amusic > Non-recovered amusic** | **A** | Right Inferior Temporal Gyrus | 17 0 16 | 61962 | 5.84* |
|  |  | Right Superior Temporal Gyrus | 58 -24 3 |  |  |
|  |  | Right Middle Temporal Gyrus | 53 -41 3 |  |  |
|  |  | Right Precentral Gyrus | 53 -10 28 |  |  |
|  |  | Right Postcentral Gyrus | 58 -21 32 |  |  |
|  |  | Right Inferior Frontal Gyrus | 53 9 9 |  |  |
|  |  | Right Putamen | 26 -10 6 |  |  |
|  |  | Right Caudate | 13 9 5 |  |  |
|  |  | Right Insula | 34 -12 17 |  |  |
|  |  | Right Thalamus | 16 -21 17 |  |  |
|  |  | Right Hippocampus | 22 -30 -5 |  |  |
| **Non-amusic > Recovered amusic** | **B** | Right Postcentral Gyrus | 23 -35 50 | 6423 | 4.73* |
|  |  | Right Precentral Gyrus | 38 -30 64 |  |  |
|  |  | Right Inferior Parietal Lobule | 34 -52 56 |  |  |
| *p < 0.005 FWE-corrected at the cluster level | | | | | |

Supplementary Table 3 Grey matter volume decreases (6-month stage – acute stage) in pitch amusia: Turku cohort.

| **6 MONTHS > ACUTE** | | | | | |
| --- | --- | --- | --- | --- | --- |
| **Condition** | **Figure 3 panel** | **Area name** | **Coordinates** | **Cluster size** | **t-value** |
| **Non-amusic > Non-recovered amusic** | **A** | Right Caudate | 15 5 21 | 61511 | 5.61** |
|  |  | Right Insula (BA 13) | 43 -1 -4 |  |  |
|  |  | Right Inferior Frontal Gyrus (BA 44, 47) | 52 22 22 |  |  |
|  |  | Right Middle Frontal Gyrus (BA 8, 11, 46, 47) | 52 49 -7 |  |  |
|  |  | Right Superior Temporal Gyrus (BA 22, 38) | 49 -3 0 |  |  |
|  |  | Right Heschl’s Gyrus (BA 41) | 52 -19 10 |  |  |
|  |  | Right Precentral Gyrus (BA 6) | 65 0 24 |  |  |
|  |  | Right Hippocampus | 27 -10 -19 |  |  |
|  |  | Right Amygdala | 27 5 -20 |  |  |
| **Non-amusic > Recovered amusic** | **B** | Right Thalamus | 4 -12 0 | 4098 | 4.79* |
|  |  | Right Caudate | 19 -14 23 |  |  |
|  |  | Right Inferior Parietal Lobule (BA 40) | 59 -41 39 | 2877 | 3.84* |
| *p < 0.05 FWE-corrected at the cluster level | | | | | |
| **p < 0.005 FWE-corrected at the cluster level | | | | | |
| BA = Brodmann area | | | | | |

Supplementary Table 4 White matter volume decreases (6-month stage – acute stage) in pitch amusia: Turku cohort.

| **6 MONTHS > ACUTE** | | | | | |
| --- | --- | --- | --- | --- | --- |
| **Condition** | **Supplementary Figure 4 panel** | **Area name** | **Coordinates** | **Cluster size** | **t-value** |
| **Non-amusic > Non-recovered amusic** | **A** | Right Caudate | 17 0 16 | 58608 | 6.16* |
|  |  | Right Superior Temporal Gyrus | 52 -31 7 |  |  |
|  |  | Right Middle Temporal Gyrus | 50 -23 -9 |  |  |
|  |  | Right Inferior Temporal Gyrus | 56 -27 -17 |  |  |
|  |  | Right Insula | 35 -21 18 |  |  |
|  |  | Right Precentral Gyrus | 55 -3 23 |  |  |
|  |  | Right Postcentral Gyrus | 56 -16 17 |  |  |
|  |  | Right Inferior Frontal Gyrus | 23 25 -16 |  |  |
|  |  | Right Parahippocampal Gyrus | 27 -28 -17 |  |  |
|  |  | Right Hippocampus | 33 -28 -7 |  |  |
|  |  | Right Midbrain | 10 -16 -19 |  |  |
|  |  | Right Putamen | 28 14 3 |  |  |
|  |  | Right Globus Pallidus | 19 -4 -2 |  |  |
|  |  | Right Thalamus | 9 -4 6 |  |  |
| **Non-amusic > Recovered amusic** | **B** | Right Insula | 30 -21 15 | 12395 | 4.75* |
|  |  | Right Inferior Parietal Lobule | 30 -50 40 |  |  |
|  |  | Right Precuneus | 19 -50 40 |  |  |
|  |  | Right Superior Temporal Gyrus | 54 -36 14 |  |  |
|  |  | Right Putamen | 27 -12 10 |  |  |
|  |  | Right Caudate | 19 6 24 | 4851 | 3.86* |
|  |  | Right Globus Pallidus | 19 -4 7 |  |  |
| *p < 0.005 FWE-corrected at the cluster level | | | | | |

Supplementary Table 5 Grey matter volume decreases (6-month stage – acute stage) in rhythm amusia: Turku cohort.

| **6 MONTHS > ACUTE** | | | | | |
| --- | --- | --- | --- | --- | --- |
| **Condition** | **Supplementary Figure 5 panel** | **Area name** | **Coordinates** | **Cluster size** | **t-value** |
| **Non-amusic > Non-recovered amusic** | **A** | Right Middle Temporal Gyrus (BA 38) | 40 6 -41 | 37033 | 5.33** |
|  |  | Right Superior Temporal Gyrus (BA 22, 38) | 38 14 -39 |  |  |
|  |  | Right Fusiform Gyrus (BA 36) | 28 -6 -39 |  |  |
|  |  | Right Inferior Frontal Gyrus (BA 9, 44, 45, 47) | 61 10 7 |  |  |
|  |  | Right Parahippocampal Gyrus (BA 35) | 28 -28 -23 |  |  |
|  |  | Right Hippocampus | 24 -5 -20 |  |  |
|  |  | Right Amygdala | 25 -3 -23 |  |  |
|  |  | Right Caudate | 11 14 9 |  |  |
| **Non-amusic > Recovered amusic** | **B** | Right Postcentral Gyrus (BA 1, 2) | 46 -30 41 | 5430 | 4.23** |
|  |  | Right Inferior Parietal Lobule (BA 40) | 43 -32 41 |  |  |
|  |  | Left Precuneus (BA 7, 31) | -12 -50 40 | 3612 | 3.87* |
|  |  | Left Inferior Parietal Lobule (BA 40) | -39 -55 41 |  |  |
| *p < 0.05 FWE-corrected at the cluster level | | | | | |
| **p < 0.005 FWE-corrected at the cluster level | | | | | |
| BA = Brodmann area | | | | | |

Supplementary Table 6 White matter volume decreases (6-month stage – acute stage) in rhythm amusia: Turku cohort.

| **6 MONTHS > ACUTE** | | | | | |
| --- | --- | --- | --- | --- | --- |
| **Condition** | **Supplementary Figure 6 panel** | **Area name** | **Coordinates** | **Cluster size** | **t-value** |
| **Non-amusic > Non-recovered amusic** | **A** | Midbrain and pons | -11 -33 -41 | 8455 | 4.84* |
|  |  | Left Cerebellum | -19 -47 -44 |  |  |
|  |  | Right Cerebellum | 22 -34 -33 |  |  |
|  |  | Right Fusiform Gyrus | 30 -50 -6 | 10162 | 4.10* |
|  |  | Right Inferior Temporal Gyrus | 41 -5 -32 |  |  |
|  |  | Right Parahippocampal Gyrus | 36 -18 -20 |  |  |
|  |  | Right Hippocampus | 30 -25 2 |  |  |
|  |  | Right Thalamus | 23 -32 7 |  |  |
|  |  | Right Caudate | 8 5 2 |  |  |
| **Non-amusic > Recovered amusic** | **B** | Right Postcentral Gyrus | 33 -27 49 | 6933 | 4.61* |
|  |  | Right Paracentral Lobule | 28 -26 59 |  |  |
| *p < 0.005 FWE-corrected at the cluster level | | | | | |

Supplementary Figure 1 Grey matter VBM results of Turku patients: amusia.

Time (6 months > Acute) x Group interactions for grey matter volume. (A) Non-amusic vs. non-recovered amusics; (B) Non-amusic vs. recovered amusics; (C) Recovered vs. non-recovered amusics. Results are thresholded at a whole-brain uncorrected p < 0.005 at the voxel level (extent threshold: k > 100 voxels) using MNI coordinates. Only clusters surviving an FWE-corrected *p* < 0.05 threshold are reported and labelled (see also Supplementary Table 1). Bar plots for GMV differences in 6 months – Acute in significant clusters (Supplementary Table 1) are shown: bar = mean, error-bar = standard error of the mean. CAU = caudate, HG = Heschl's gyrus, IFG = inferior frontal gyrus, INS = insula, IPL = inferior parietal lobule, MFG = middle frontal gyrus, MTG = middle temporal gyrus, PCG = postcentral gyrus, PreCG = precentral gyrus, PUT = putamen, STG = superior temporal gyrus.


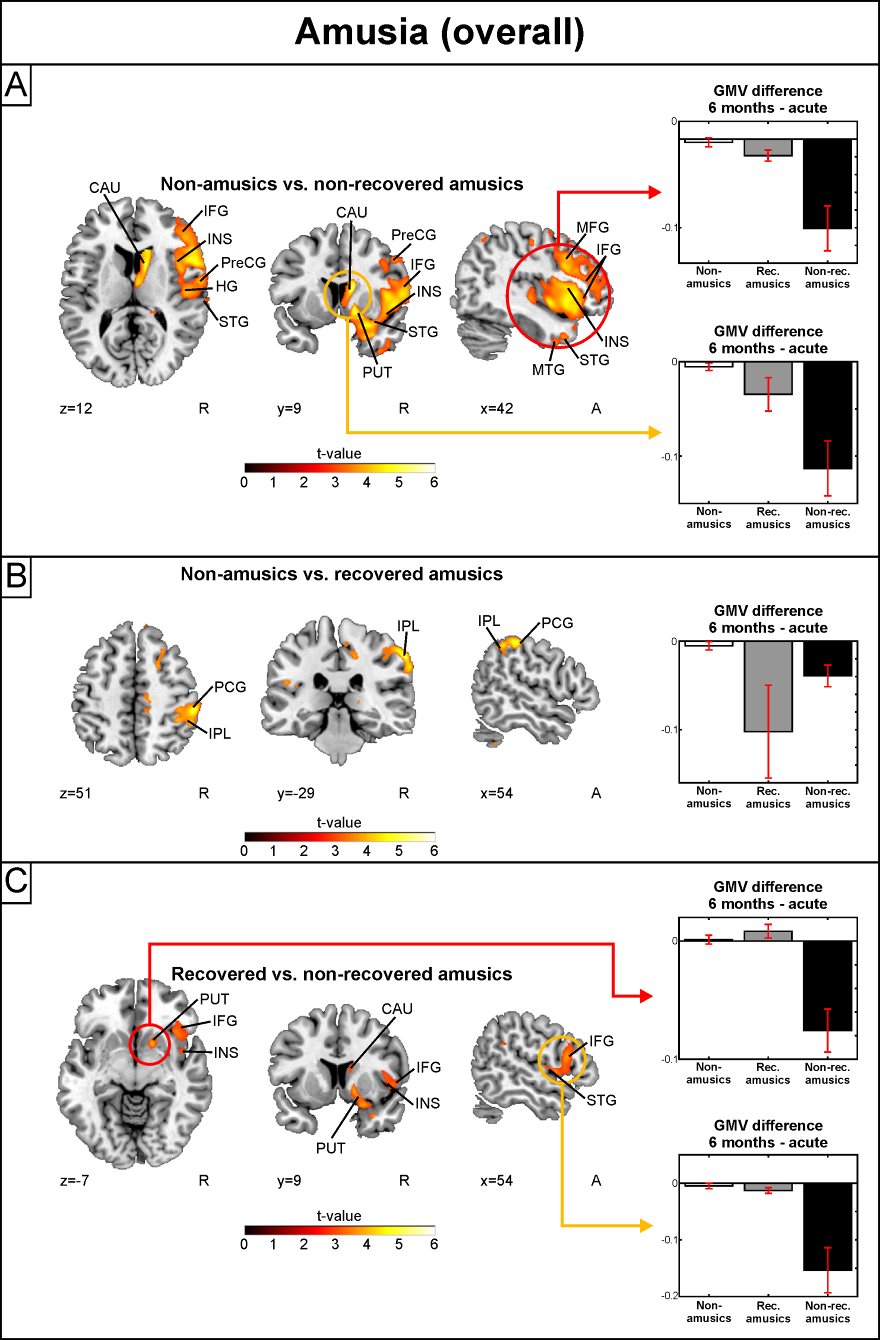


Supplementary Figure 2 White matter VBM results of Turku patients: amusia.

Time (6 months > Acute) x Group interactions for white matter volume. (A) Non-amusic vs. non-recovered amusics; (B) Non-amusic vs. recovered amusics. Results are thresholded at a whole-brain uncorrected p < 0.005 at the voxel level (extent threshold: k > 100 voxels) using MNI coordinates. Only clusters surviving an FWE-corrected *p* < 0.05 threshold are reported and labelled (see also Supplementary Table 2). Bar plots for GMV differences in 6 months – Acute in significant clusters (Supplementary Table 2) are shown: bar = mean, error-bar = standard error of the mean. CAU = caudate, HIP = hippocampus, IFG = inferior frontal gyrus, INS = insula, IPL = inferior parietal lobule, MTG = middle temporal gyrus, PCG = postcentral gyrus, PUT = putamen, PreCG = precentral gyrus, STG = superior temporal gyrus, THA = thalamus.


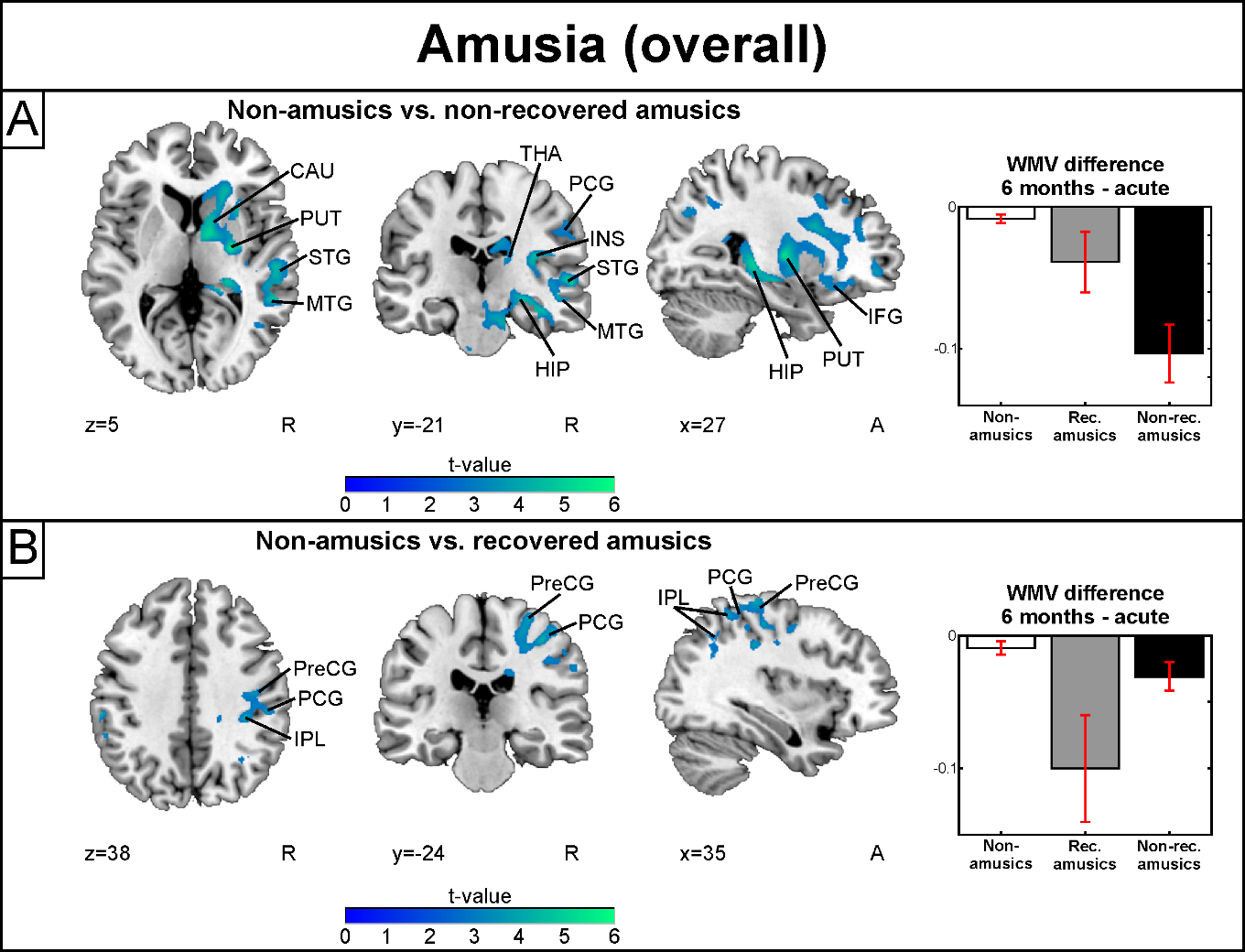


Supplementary Figure 3 Grey matter VBM results of Turku patients: pitch amusia.

Time (6 months > Acute) x Group interactions for grey matter volume. (A) Non-amusic vs. non-recovered amusics; (B) Non-amusic vs. recovered amusics. Results are thresholded at a whole-brain uncorrected p < 0.005 at the voxel level (extent threshold: k > 100 voxels) using MNI coordinates. Only clusters surviving an FWE-corrected *p* < 0.05 threshold are reported and labelled (see also Supplementary Table 3). Bar plots for GMV differences in 6 months – Acute in significant clusters (Supplementary Table 3) are shown: bar = mean, error-bar = standard error of the mean. AMY = amygdala, CAU = caudate, HG = Heschl’s gyrus, HIP = hippocampus, IFG = inferior frontal gyrus, INS = insula, IPL = inferior parietal lobule, MFG = middle frontal gyrus, PreCG = precentral gyrus, STG = superior temporal gyrus, THA = thalamus.


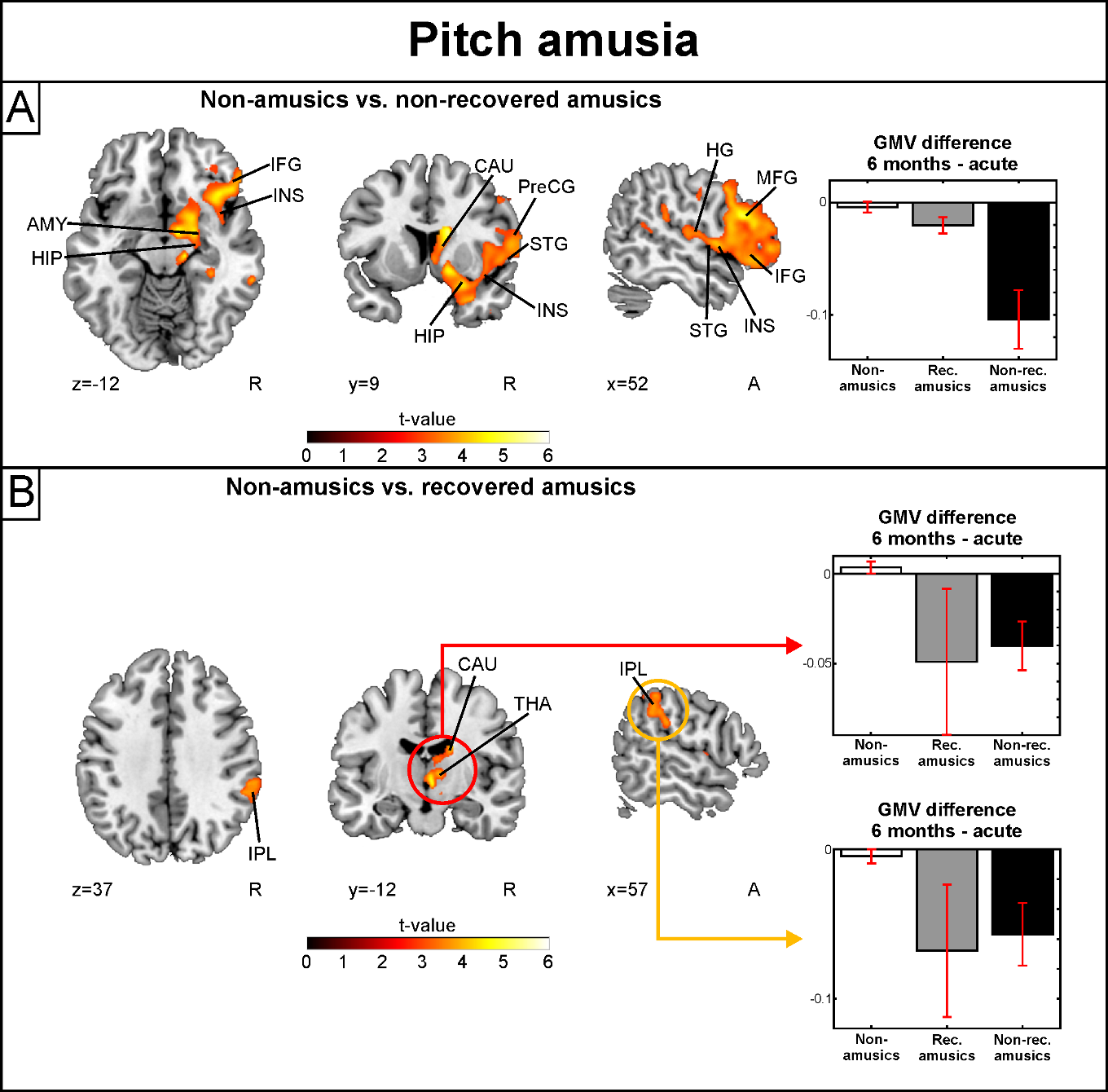


Supplementary Figure 4 White matter VBM results of Turku patients: pitch amusia.

Time (6 months > Acute) x Group interactions for white matter volume. (A) Non-amusic vs. non-recovered amusics; (B) Non-amusic vs. recovered amusics. Results are thresholded at a whole-brain uncorrected p < 0.005 at the voxel level (extent threshold: k > 100 voxels) using MNI coordinates. Only clusters surviving an FWE-corrected *p* < 0.05 threshold are reported and labelled (see also Supplementary Table 4). Bar plots for GMV differences in 6 months – Acute in significant clusters (Supplementary Table 4) are shown: bar = mean, error-bar = standard error of the mean. CAU = caudate, GP = globus pallidus, HIP = hippocampus, INS = insula, IFG = inferior frontal gyrus, IPL = inferior parietal lobule, ITG = inferior temporal gyrus, MTG = middle temporal gyrus, PCG = postcentral gyrus, PreCG = precentral gyrus, PUT = putamen, STG = superior temporal gyrus.


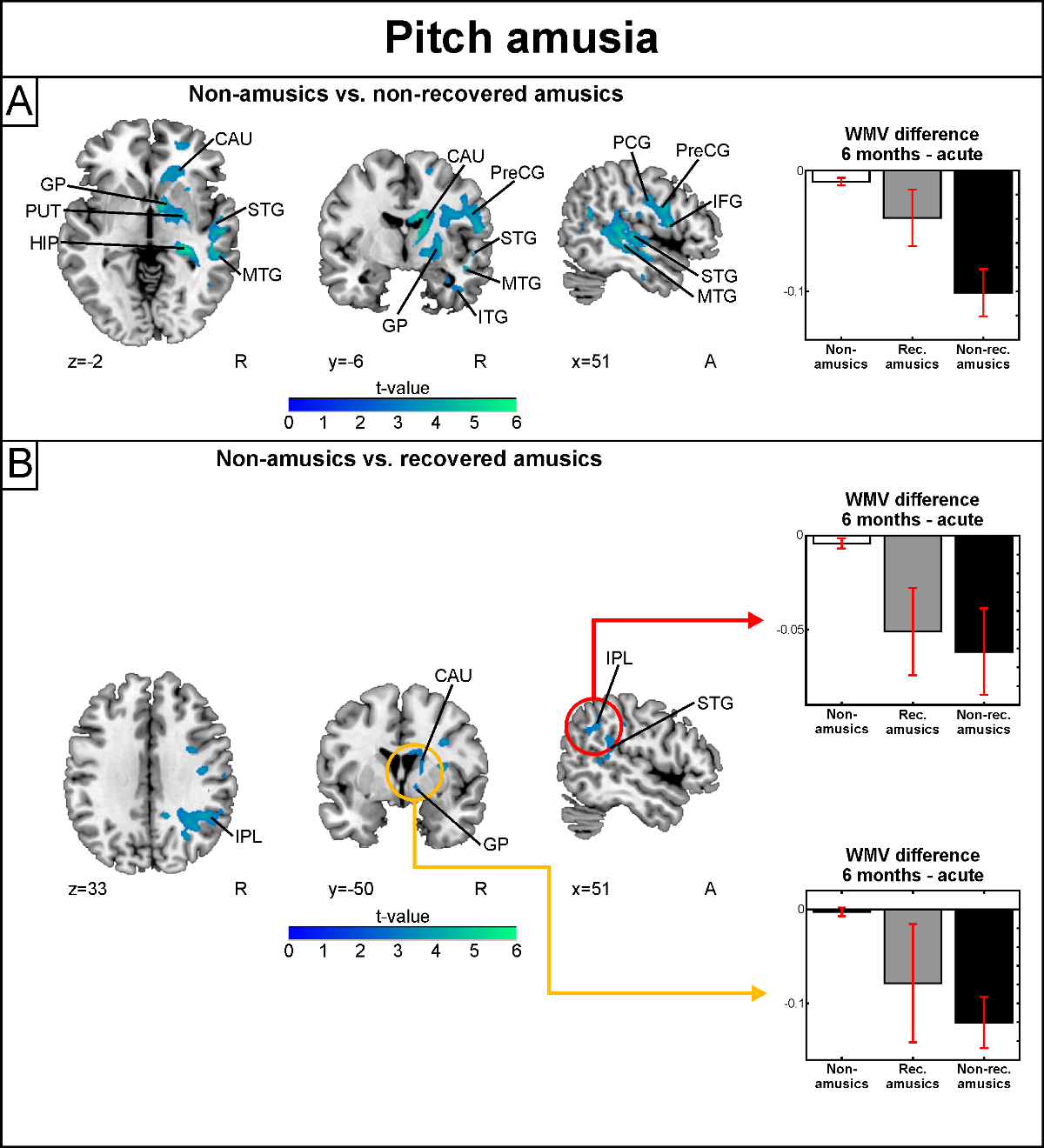


Supplementary Figure 5 Grey matter VBM results of Turku patients: rhythm amusia.

Time (6 months > Acute) x Group interactions for grey matter volume. (A) Non-amusic vs. non-recovered amusics; (B) Non-amusic vs. recovered amusics. Results are thresholded at a whole-brain uncorrected p < 0.005 at the voxel level (extent threshold: k > 100 voxels) using MNI coordinates. Only clusters surviving an FWE-corrected *p* < 0.05 threshold are reported and labelled (see also Supplementary Table 5). Bar plots for GMV differences in 6 months – Acute in significant clusters (Supplementary Table 5) are shown: bar = mean, error-bar = standard error of the mean. CAU = caudate, IFG = inferior frontal gyrus, INS = insula, IPL = inferior parietal lobule, PCG = postcentral gyrus, STG = superior temporal gyrus.


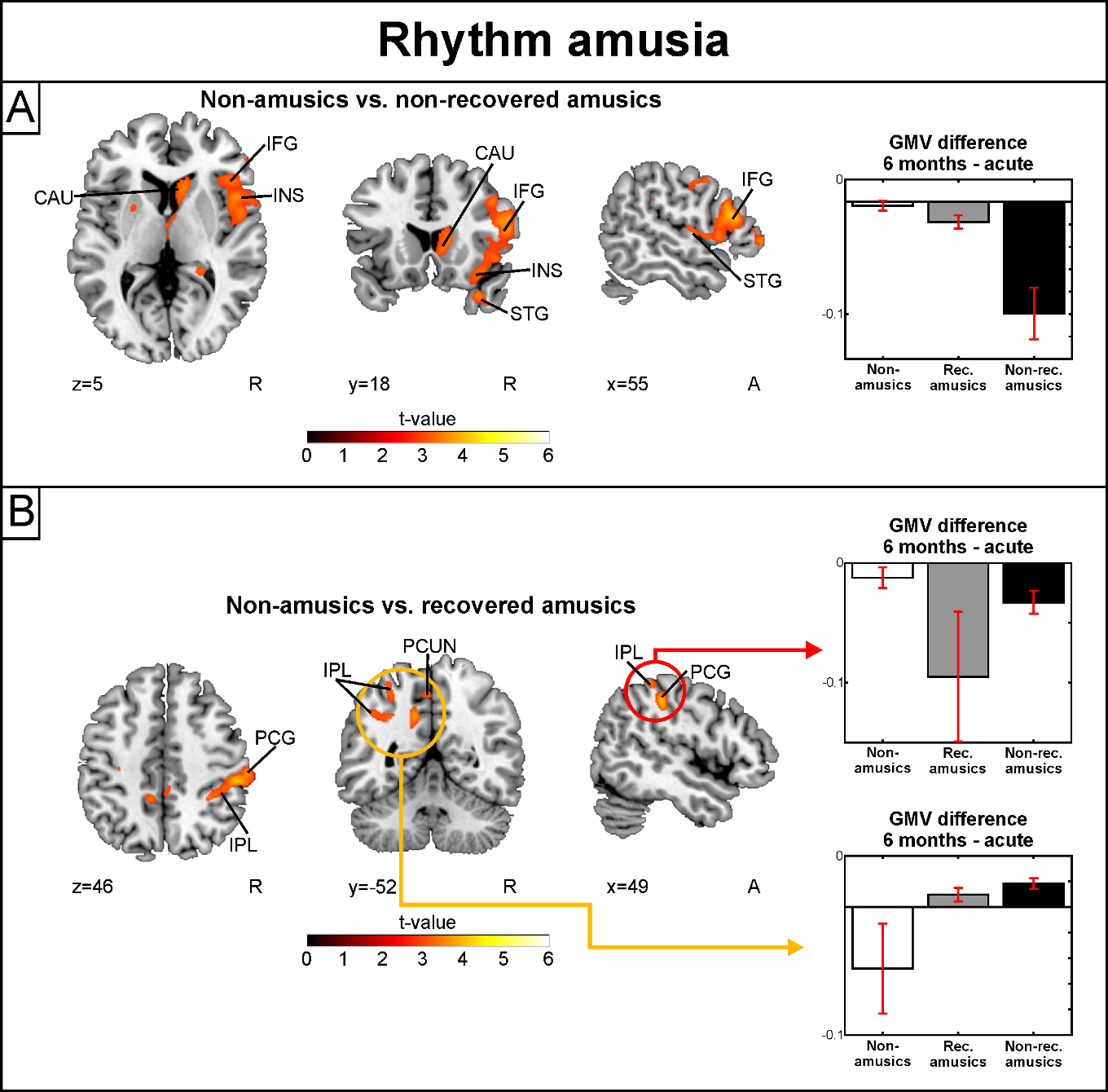


Supplementary Figure 6 White matter VBM results of Turku patients: rhythm amusia.

Time (6 months > Acute) x Group interactions for white matter volume. (A) Non-amusic vs. non-recovered amusics; (B) Non-amusic vs. recovered amusics. Results are thresholded at a whole-brain uncorrected p < 0.005 at the voxel level (extent threshold: k > 100 voxels) using MNI coordinates. Only clusters surviving an FWE-corrected *p* < 0.05 threshold are reported and labelled (see also Supplementary Table 6). Bar plots for GMV differences in 6 months – Acute in significant clusters (Supplementary Table 6) are shown: bar = mean, error-bar = standard error of the mean. FG = fusiform gyrus, IPL = inferior parietal lobule, ITG = inferior temporal gyrus, MB = midbrain, PCL = paracentral lobule, PCG = postcentral gyrus.


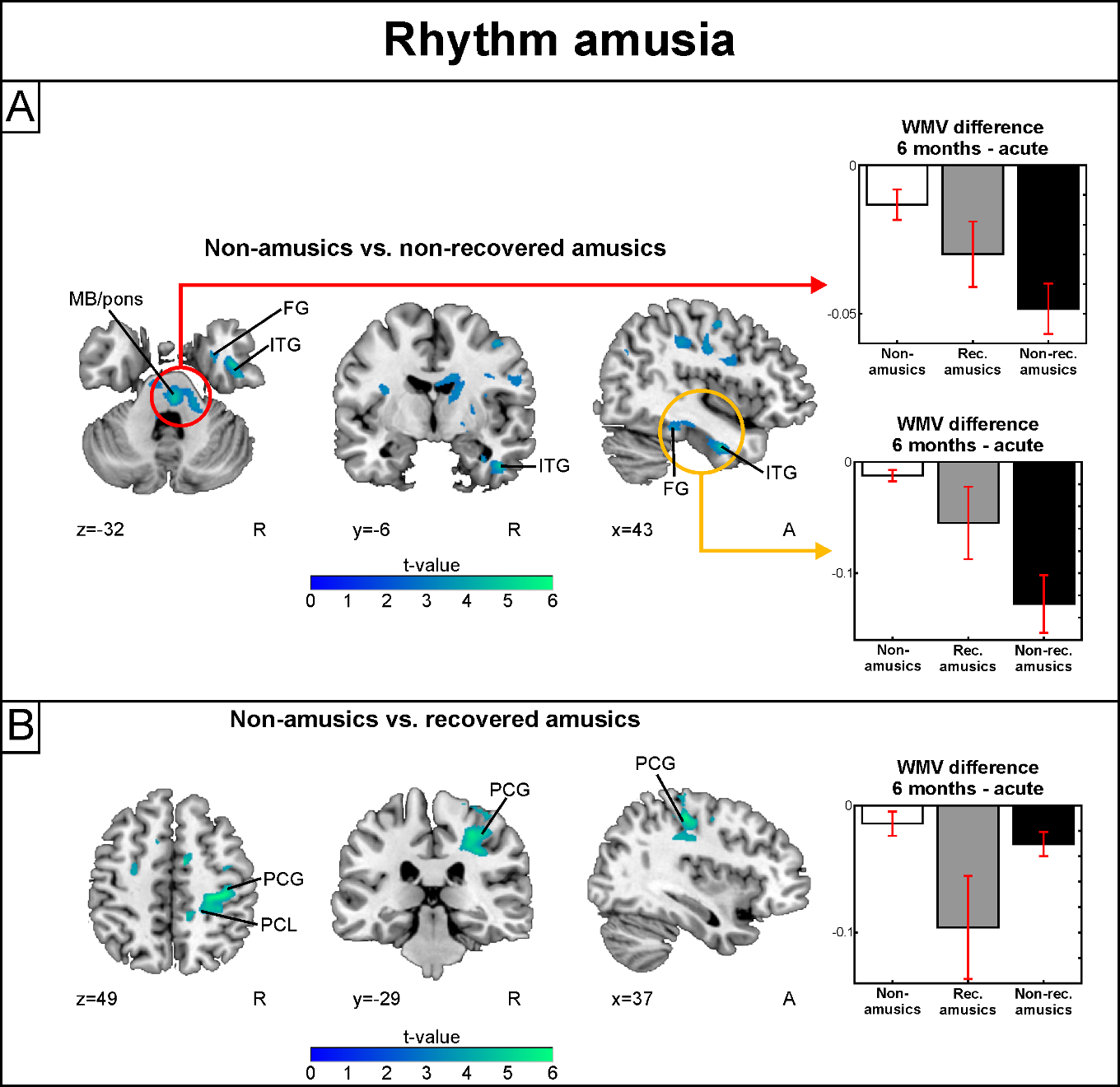

Supplement: Supplementary file 1 [file DataSheet1.doc]
